# Lipopeptides and antibiotics from a marine *Bacillus pumilus* mediate a potential “catch and kill” effect on pathogenetic *Vibrio parahaemolyticus*

**DOI:** 10.1128/msystems.01440-25

**Published:** 2025-12-31

**Authors:** Hilary J. Ranson, Yan-Song Ye, Valentina Z. Petukhova, Abigail Green-Saxena, Ruolin He, Jiadong Sun, Bhaskar Godugu, Laura M. Sanchez, Qihao Wu, David C. Rowley

**Affiliations:** 1 Department of Biomedical & Pharmaceutical Sciences, The University of Rhode Island, Kingston, Rhode Island, USA; 2 Department of Pharmaceutical Sciences, University of Pittsburgh 6614, Pittsburgh, Pennsylvania, USA; 3 Department of Pharmaceutical Sciences, University of Illinois Chicago 14681, Chicago, Illinois, USA; 4 Bioinformatics Group, Wageningen University 593528 , Wageningen, the Netherlands; 5 Department of Chemistry, University of Pittsburgh 6614, Pittsburgh, Pennsylvania, USA; 6 Department of Chemistry and Biochemistry, University of California Santa Cruz 8787, Santa Cruz, California, USA; Academia Sinica Agricultural Biotechnology Research Center, Tainan City, Taiwan

**Keywords:** marine *Bacillus*, lipopeptides, antibiotics, MALDI TOF IMS

## Abstract

Bacteria produce a diverse range of specialized metabolites that influence the health and behavior of neighboring cells and, therefore, have potential applications in treating diseases. Deciphering the intended ecological functions of specialized metabolites is challenging due to the small scales at which these interactions occur and the complexity of unraveling simultaneous responses to multiple signals. In this study, we investigated the chemical interactions between two marine bacterial colonies, *Vibrio parahaemolyticus* PSU5429 and *Bacillus pumilus* YP001. When the two bacteria were grown in proximity on agar, *V. parahaemolyticus* exhibited swarming motility toward *B. pumilus*, but close approach to the *B. pumilus* colony was impeded by a zone of inhibition. Matrix-assisted laser desorption/ionization time-of-flight imaging mass spectrometry (MALDI-TOF IMS) suggested that lipopeptides produced by *Bacillus* induced swarming motility, a finding corroborated by genomic and chemical analyses of YP001. Based on activity and metabolomics guidance, the antibiotic amicoumacin B was found to be responsible for the observed antibiosis, while swarming motility by *V. parahaemolyticus* was induced by lipopeptides and two lipoamides. In this scenario, lipopeptide production by the *Bacillus* colony induces the *Vibrio* colony to swarm toward a lysis zone, resulting in a possible “catch and kill” effect. These results demonstrate the complexity of behaviors and outcomes exhibited by microbes under the simultaneous influence of different allelochemicals, suggesting possible interplays between antibiotics and compounds that induce motility.

**IMPORTANCE:**

Microbes communicate and compete using small molecules, yet linking specific metabolites to visible behaviors is difficult. We combine imaging mass spectrometry, genomics, analytical chemistry, and bioassays to decode an interaction between a marine *Bacillus* and the pathogen *Vibrio parahaemolyticus*. Surfactin-like lipopeptides act at a distance to stimulate *Vibrio* swarming and draw cells toward the colony. Amicoumacin B accumulates at the interface and halts growth, yielding a simple “catch and kill” outcome. This study shows that the spatial localization of natural products shapes microbial behavior on surfaces and provides a general, scalable workflow that maps chemistry to phenotype. Beyond this case, the approach can be applied broadly to understand and, ultimately, tune microbial interactions relevant to marine ecosystems, aquaculture health, and microbiome engineering.

## INTRODUCTION

Bacteria use chemical communication to coordinate gene expression and synchronize behaviors, such as bioluminescence, virulence factor production, and biofilm formation (1). The secretion of specialized metabolites can also influence competitive outcomes in mixed communities (2), for example, antibiotics that limit growth of neighboring cells (3), metallophores that chelate metals (4–6), and compounds that modulate motility (7). As an example of the latter, members of the genus *Bacillus* are known to secrete surfactins that modulate surface tension, enabling swarming motility (8). These biosynthetic processes are energetically expensive and ineffective for individual cells but become advantageous when performed collectively in a population. Deciphering the intended ecological functions of specialized metabolites is challenging due to the small scales at which these interactions occur and the complexity of unraveling simultaneous responses to multiple signals.

Imaging mass spectrometry (IMS) is a particularly useful technique for observing chemically mediated microbial interactions on surfaces such as agar (9). IMS methods, such as matrix-assisted laser desorption/ionization time-of-flight (MALDI-TOF), provide a spatial map of ionizable molecules as they diffuse away from a source, such as a microbial colony (10–12). Mapping the metabolic exchanges between groups of cells can provide insights into the ecological functions of specialized metabolites and aid in the formulation of hypotheses for how compounds influence competitive outcomes (13–16).

Bacteria belonging to the genus *Bacillus* are recognized as prolific producers of allelochemicals, especially biologically active peptides. For example, the antibiotics polymyxin B (17) and colistin (18) are examples of medically beneficial peptide drugs derived from *Bacillus* species. Surfactins are cyclic lipopeptides that reduce surface tension and facilitate coordinated swarming, a form of social motility observed in many *Bacillus* species (19). Surfactins have also been implicated in antibiosis toward other microbes, initiation of biofilm production, surface attachment, and functioning as a chemoattractant (20, 21). MALDI-TOF IMS is an *in situ* method particularly well suited for the detection and characterization of secreted peptides and has proven useful in prior studies of chemically mediated microbial interactions between *Bacillus* and other bacteria (22, 23).

Our interest in bacterial interactions in marine aquaculture settings led us to investigate the chemical interplay between *Bacillus pumilus* YP001 and *Vibrio parahaemolyticus* PSU5429, a marine pathogen of significant importance in aquaculture. When co-localized on an agar surface, the *Vibrio* colony was observed to commence swarming motility in the direction of the *Bacillus* colony. As the vibrios approached the *Bacillus* colony, a lysis zone became visible, suggesting that both attraction and antibiosis were acting upon the motile vibrios. To better understand how metabolic exchanges drive this microbial interaction, we combined MALDI-TOF IMS, genome analysis, and studies of purified compounds. Here, we demonstrate how multiple compounds act in concert to combine chemoattractant and antibiosis effects on a community of *V. parahaemolyticus*.

## RESULTS

**Fig 1:**
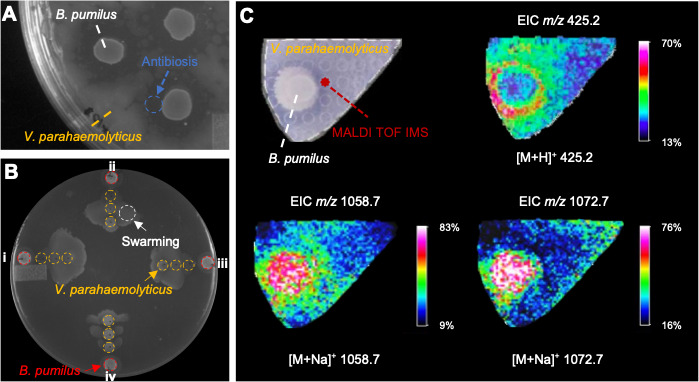
MALDI-IMS-based chemical profiling of the interaction zone between *B. pumilus* YP001 and *V. parahaemolyticus* PSU5429. (**A**) Growth inhibition of *B. pumilus* YP001 against PSU5429 after 24 h of incubation on YP30 at 27°C. The antibiosis effect zone is indicated by a dashed circle. (**B**) Swarming motility assay for *B. pumilus* YP001 (red dashed circle) plated in co-culture with *V. parahaemolyticus* PSU5429 (yellow dashed circles) at concentrations of 1 × 10^5^ CFU/mL (left, I), 1 × 10^2^ CFU/mL (top, ii), 1 × 10^1^ CFU/mL (right, iii), and no dilution (bottom, iv). The plate was imaged after 24 h of incubation at 27°C. The swarming motility effect is indicated by a white dashed circle. *V. parahaemolyticus* PSU5429 was inoculated as three separate colonies. (**C**) MALDI-IMS imaging shows the spatial distribution of compounds produced during the chemical interaction of *B. pumilus* YP001 and *V. parahaemolyticus* PSU5429. Molecular features were putatively identified as amicoumacin B {*m/z* 425.2 [M+H]^+^} and two surfactins {*m/z* 1058.7 [M+Na]^+^, and *m/z* 1072.7 [M+Na]^+^}. Extracted ion images showed that amicoumacin B was localized within the antibiosis zone, while surfactins were primarily distributed within or adjacent to the *B. pumilus* YP001 colony.

During a binary screening program aimed at identifying marine bacteria exhibiting antibiosis against *V. parahaemolyticus* PSU5429, we discovered that *B. pumilus* YP001, a bacterium isolated from a female American lobster (*Homarus americanus*) carrying eggs in Narragansett Bay, RI, produced colonies that created zones of inhibition when grown on lawns of PSU5429 (Fig. 1A) and other *Vibrio* pathogens (Fig. S1). To further explore this interaction, we spotted colonies of YP001 and PSU5429 in proximity on YP30 agar plates. Following incubation, swarming motility was observed for the *V. parahaemolyticus* in the direction of the *Bacillus* colony (Fig. 1B). However, at approximately 8 mm, the motile PSU5429 became impeded from approaching YP001 (Fig. 1A), suggesting the concomitant production of an antibiotic compound(s).

We hypothesized that specialized metabolites were responsible for inducing swarming motility by *Vibrio* and the lysis zone surrounding the *B. pumilus* colony. To visualize the secretion of specialized metabolites by the interacting colonies on thin agar plates, we implemented a MALDI-TOF IMS approach, motivated by previous successful applications of this approach in investigations of specialized metabolism by *Bacillus* spp. MALDI-TOF IMS was conducted in positive ion mode, producing a 2D spatial map of chemical interactions between the two bacterial colonies (Fig. 1C) at the 24-hour time point, when pronounced *Vibrio* swarming and antibiotic effects were observed. Prominent features included compounds with *m/z* 1058.7 [M+Na]^+^ and 1072.7 [M+Na]^+^ extending from the *B. pumilus* colony to where *V. parahaemolyticus* was swarming (Fig. 1C; Fig. S2 and S3). The masses of the two chemical features were consistent with lipopeptides, such as surfactins, compounds that promote swarming motility in phylogenetically diverse bacteria, including *B. subtilis*, *Pseudomonas* spp., *V. parahaemolyticus*, and *Serratia* spp. (24). Other IMS features included ions localized within the zone of inhibition, signaling a potential role in the observed antibiosis (Fig. 1C; Fig. S2 and S4). Prominent ions included those with *m/z* 425.2 [M+H]^+^ (Fig. 1C). Based on a review of antibiotic compounds produced by *Bacillus* species, the ion at *m/z* 425.2 was consistent with the expected [M+H]^+^ of amicoumacin B, an antibacterial compound produced by a hybrid PKS-NRPS biosynthetic pathway previously identified in *Bacillus* spp (25).

To investigate the biosynthetic potential of *B. pumilus* YP001 and possibly identify compounds consistent with the IMS results, whole-genome sequencing of strain YP001 was performed. A draft genome from YP001 was assembled and comprised 14 contigs, averaging 3,640,419 bp in size, with an average GC content of 53.5%. The genome was annotated with the Rapid Annotation Subsystem Technology (RAST) server (26), which identified 3,797 open reading frames. The genome was next analyzed using antiSMASH (Antibiotics and Secondary Metabolite Analysis Shell) (27) to identify biosynthetic gene clusters (BSGs) involved in specialized metabolite production. Overall, 12% of the genome (i.e., 427,1038 nt) was broadly characterized as being involved in specialized metabolite biosynthesis. The genome of *B. pumilus* YP001 encoded 11 BGCs, including three non-ribosomal peptide synthase (NRPS) clusters, two terpene biosynthesis pathways, type 1 polyketide synthase genes, and one hybrid NRPS cluster.

Analysis of an NRPS cluster indicated that YP001 was capable of producing lipopeptides (Fig. S5). Although antiSMASH annotated the putative lipopeptide BGC in region 3.1 as a predicted lichenysin cluster, the mass spectrometry data did not match any reported lichenysin molecular features. A more detailed analysis of this region revealed key features suggesting divergence from established lichenysin pathways. Closer inspection of region 3.1 in strain YP001 showed that the adenylation domain of module 1 was predicted to activate glutamic acid (Glu) (Fig. S5), a critical distinction from lichenysin biosynthesis, which incorporates glutamine (Gln) at this position (Fig. S5). This residue specificity aligns more with surfactins, which also utilize Glu as the initiating amino acid, suggesting that strain YP001 may produce a new surfactin-like lipopeptide rather than a lichenysin. We also noted the absence of genes encoding NRPS modules responsible for assembling the Val-Asp-D-Leu motif in surfactin, such as *srfAB* in *Bacillus velezensis* FZB42 and *C6X96_RS144990* in *B. pumilus* NMSW10 (Fig. S5). A thorough genome-wide search identified region 9.1, which showed 73% sequence similarity to *C6X96_RS144990* (Fig. S6). Although analysis of this gene suggested that module 6, which installs D-Leu, was missing, this module is typically conserved across surfactin- and lichenysin-type lipopeptides (Fig. S5). Furthermore, MALDI-TOF IMS of the two features (*m/z* 1058.7, [M+Na]^+^ and *m/z* 1072.7, [M+Na]^+^; Fig. S3) supported the incorporation of Leu. Taken together, the genomic and MALDI-TOF IMS evidence provided a tentative assignment of the two compounds as surfactins. Alignment of YP001 genes with *amiA* through *amiM* from KCTC 13429 and other known producer strains, including *B. pumilus* SF214, *B. pumilus* SH-B9, and *B. subtilis* fmb60, showed strong homology among the clusters (Fig. S7). This supports region 4.1 as the putative amicoumacin BGC, and the detected metabolite feature in YP001 is consistent with amicoumacin B (28).

**Fig 2:**
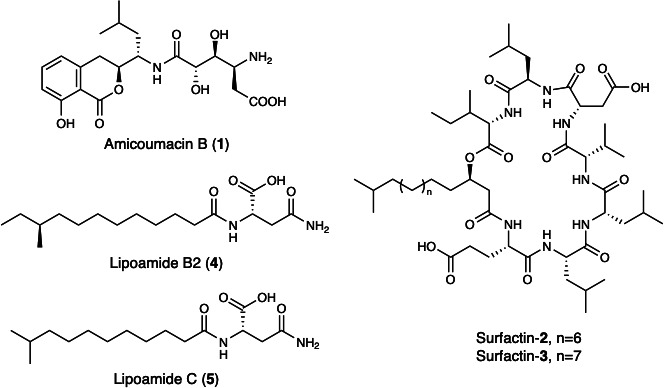
Chemical structures of compounds **1–5**. Amicoumacin B (**1**) exhibited antibacterial activity against *V. parahaemolyticus* PSU5429. Surfactin-**2** and surfactin-**3** strongly induced swarming in both *V. parahaemolyticus* PSU5429 and *V. coralliilyticus* RE22. Lipoamide B2 (**4**) weakly promoted swarming in *V. parahaemolyticus* PSU5429, whereas lipoamide C (**5**) was inactive. The chemical structures of amicoumacin B and lipoamides were elucidated by HR-ESI-MS and 2D NMR analyses, whereas surfactins were tentatively identified based on HR-ESI-MS data and BGC analysis.

The results from the MALDI-TOF IMS experiments and genome analysis encouraged the structure characterization of bioactive compounds produced by *B. pumilus* YP001 to validate their biosynthesis and bioactivities. Extraction from agar plates would require hundreds of plates to obtain sufficient material for the isolation and confirmation of bioactive compounds. Therefore, we first explored whether monoculture of *B. pumilus* in liquid medium could support large-scale preparation of bioactive compounds. To test this, *B. pumilus* was cultured in YP30 (yeast extract, peptone, and sea salt) medium supplemented with Amberlite XAD-16 resin. The resin eluate from this monoculture recapitulated the antibiosis and *Vibrio* swarming phenotypes observed during co-incubation on LB agar. The resulting active extracts were fractionated by C18 reversed-phase chromatography, and compound isolation was guided by MS as well as bioassays against *V. parahaemolyticus* PSU5429. Based on the observed zones of growth inhibition in disk diffusion assays, fractions containing a chemical feature with an *m/z* of 425.2 [M+H]^+^ were found to exhibit antibiotic activity. Subsequent isolation, together with *de novo* two-dimensional NMR and high-resolution ESI-MS, confirmed the production of amicoumacin B (**1**, Fig. 2; Table S1; Fig. S8 to S13). Regarding the swarming activity, bioassay-guided fractionation resulted in the isolation of two lipopeptides, whose masses matched the MALDI-TOF IMS features observed on the plates (Fig. S14 to S17). Two-dimensional NMR spectra were consistent with lipopeptide structures but provided limited structural detail due to repetitive amino acid motifs that produced significant signal overlap. By integrating MALDI-TOF IMS, high-resolution ESI-MS, and genome analysis of the NRPS BGC, we tentatively assigned the structures shown in Fig. 2. Amino acid configurations were inferred from NRPS domain architecture, including A-domain specificities and epimerization modules. Due to the inconsistent naming of surfactin congeners across chain lengths and amino acid variants in the literature, we refer to these compounds as surfactin-**2** and surfactin-**3** in this study (Fig. 2).

**Fig 3:**
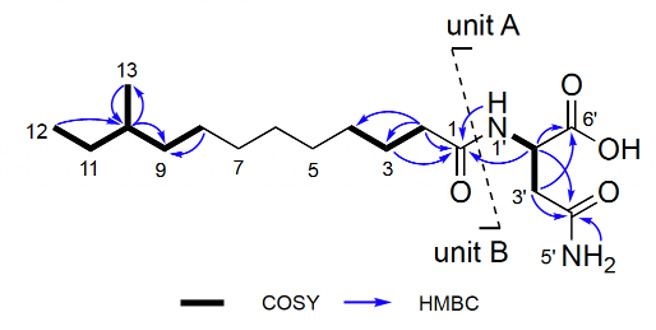
Structural elucidation of lipoamide B2 (**4**) is shown. ^1^H−^1^H COSY and key HMBC correlations for lipoamide B2 are indicated.

Interestingly, two additional compounds were isolated from the swarming-active fractions. These compounds were identified as non-lipopeptides based on their distinct NMR and MS profiles. The purified isolates underwent *de novo* structural elucidation, resulting in the identification of lipoamides B2 and C (**4** and **5**, Fig. 2). Lipoamide B2 (**4**) was obtained as an optically active, colorless oil {$[\alpha {]}_{D}^{20}$ −8.43 (*c* 0.1, MeOH)}. The HRESIMS analysis determined the molecular formula of **4** to be C_17_H_32_N_2_O_4_ (*m/z* 351.2226 [M+Na]^+^, calcd. 351.2254; Fig. S18), requiring three degrees of unsaturation. A characteristic IR absorption (*ν*_max_ 1663 cm^−1^, Fig. S25) indicated the presence of an amide. Its ^13^C NMR spectrum (Fig. S21; Table 1), combined with an HSQC experiment (Fig. S22), revealed 17 carbon resonances, including two methyls, 10 sp^3^ methylenes, two sp^3^ methines, and three sp^2^ quaternary carbons (three carbonyl carbons at *δ*_C_ 173.0, 172.3, and 171.9, two of which were identified as amide carbonyl carbons based on the MS and IR spectrum of **4**). The above functional groups accounted for all three degrees of unsaturation, indicating the presence of a linear structure in **4**. The existence of an asparagine unit (unit A, Fig. 3) was determined by the clear ^1^H−^1^H COSY correlations of H-1′ (*δ*_H_ 7.94, d, *J* = 7.5 Hz)/H-2′ (*δ*_H_ 4.45 m)/H_2_-3’ (*δ*_H_ 2.42, m; *δ*_H_ 2.56, m) and key HMBC correlations from these protons to C-6′ (*δ*_C_ 173.0) and C-4′ (*δ*_C_ 171.9), along with the identical HMBC correlations from H-1′ to C-2′ (*δ*_C_ 48.9) and from H_2_-5’ (*δ*_H_ 6.85, s; *δ*_H_ 7.37, s) to C-4′. Two methyl signals at *δ*_H_ 0.83 (t, *J* = 7.5 Hz) and *δ*_H_ 0.80 (d, *J* = 6.6 Hz), along with a broad signal at *δ*_H_ 1.23, strongly indicated the presence of *anteiso*-methyl-branched fatty acid chain. This was further verified by ^1^H−^1^H COSY correlations between H_3_-12/H-10 (*δ*_H_ 1.32, m) and H_2_-11 (*δ*_H_ 1.11, m; *δ*_H_ 1.32, m), H_3_-13/H_2_-9 (*δ*_H_ 1.07, m; *δ*_H_ 1.27, m), and H-10, as well as HMBC correlations from H_3_-12/H_3_-13 to C-10 and H-10 to C-9. Furthermore, the HMBC correlation of H_2_-2 (*δ*_H_ 2.07, t, *J* = 7.2 Hz) and C-1 (*δ*_C_ 171.3), bearing in mind the MS of **4**, indicated the presence of a 12-membered side chain with a C-1 acyl group and a methyl branch at the antepenultimate carbon (unit B, Fig. 3). Finally, the connection between units A and B was established by the correlations from H-1′/H-2′/H_2_-2 to C-1, allowing construction of the planar structure of **4**. The NMR spectroscopic data of compound **5** (Fig. S26 to S30) provided conclusive evidence that its structure was identical to the previously published lipoamide C (29, 30). The asparagines in both lipoamides were assigned as the L-configuration based on comparison of optical rotations {$[\alpha {]}_{D}^{23.2}$ −8.43 (*c* 0.1, MeOH) for **4** and $[\alpha {]}_{D}^{23.2}$ −7.05 (*c* 0.1, MeOH) for **5**} with the model compound *N*-acetyl-L-asparagine. For lipoamide B2 (**4**), C-10 was tentatively assigned as the *S* configuration based on its likely biogenic origin from L-isoleucine (31).

**TABLE 1:** 1_H_ and ^13^C NMR data (*δ* in ppm, J in Hz) for lipoamide B2 (**4**) in DMSO-*d*_6_.

Position	Type	δ_C_	δ_H_ (J in Hz)
1	qC	172.3	–^*a*^
2	CH_2_	35.1	2.07 t (7.2)
3	CH_2_	25.2	1.45 m
4	CH_2_	28.5	1.23 m
5	CH_2_	28.9	1.23 m
6	CH_2_	28.9	1.23 m
7	CH_2_	28.9	1.22 m
8	CH_2_	26.5	1.27 m
9	CH_2_	36.0	1.07 m, 1.27 m
10	CH	33.8	1.32 m
11	CH_2_	29.4	1.11 m, 1.32 m
12	CH_3_	11.7	0.80 t (7.5)
13	CH_3_	19.1	0.83 d (6.6)
1’	NH	–	7.94 d (7.5)
2’	CH	48.9	4.45 m
3’	CH_2_	37.0	2.42 m, 2.56 m
4'	qC	171.9	–
5’	NH_2_	–	6.85 s, 7.37 s
6’	qC	173.0	–

^*a*^ –, not applicable.

**Fig 4:**
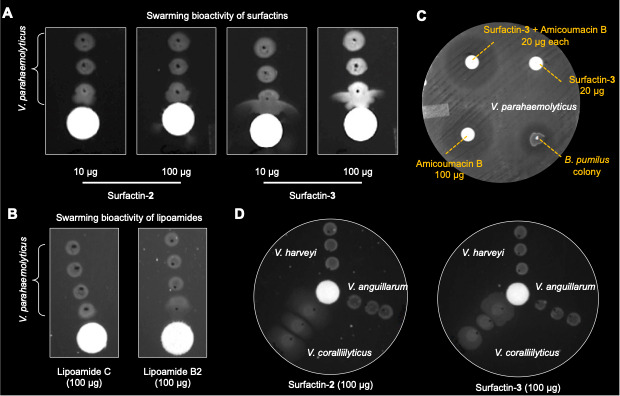
Effects of purified compounds on *V. parahaemolyticus* PSU5429 and other vibrios. (**A**) Swarming bioactivity of surfactin-**2** and surfactin-**3** against *V. parahaemolyticus* PSU5429. Surfactin-**2** (10 μg, first lane; 100 μg, second lane) and surfactin-**3** (10 μg, third lane; 100 μg, fourth lane) all induced strong swarming after 24 h of incubation at 27°C on YP agar. Surfactin-**3** showed slightly stronger activity compared to surfactin-**2**. (**B**) Swarming assay for lipoamide C (100 μg, left lane) and lipoamide B2 (100 μg, right lane) against *V. parahaemolyticus* PSU5429 after 24 h of incubation at 27°C. Only lipoamide B2 showed weak swarming activity. (**C**) Purified surfactin-**3** and amicoumacin B recapitulate the antimicrobial activity of the *B. pumilus* YP001 colony. Top left: Combination of surfactin-**3** (20 μg/disk) and amicoumacin B (20 μg/disk) exhibited strong inhibitory activity against *V. parahaemolyticus* PSU5429. Top right: Surfactin-**3** (20 μg/disk) alone showed no observable inhibitory activity against *V. parahaemolyticus* PSU5429. Bottom left: Amicoumacin B (100 μg/disk) alone exhibited inhibitory activity comparable to the combination of surfactin-**3** and amicoumacin B (20 μg/disk each). Bottom right: *B. pumilus* YP001 colony (5 μL at 1 × 10^5^ CFU/mL from a broth culture) as a control exhibited observable inhibitory activity against *V. parahaemolyticus* PSU5429. (**D**) Surfactins selectively induce swarming motility in *Vibrio coralliilyticus* RE22. Swarming assay for 100 μg of surfactin-**2** and surfactin-**3** against *Vibrio coralliilyticus* RE22, *Vibrio harveyi* BB120, and *Vibrio anguillarum* NB10Sm after 48 h of incubation at 27°C. Both surfactins induced swarming only in *V. coralliilyticus* RE22.

Finally, a motility assay was used to investigate the effects of specific compounds on the initiation of swarming by *V. parahaemolyticus* PSU5429. Consequently, *V. parahaemolyticus* was observed to swarm toward sources of highly purified surfactins or lipoamides. Close proximity of PSU5429 to either *B. pumilus* YP001 or disks loaded with lipopeptide compounds **2–5** stimulated swarming behavior but displayed no observable antibacterial effects (Fig. 4A and B). Isolated surfactin-**2** and surfactin-**3** induced strong directional swarming when loaded onto disks at 10 and 100 μg/disk (Fig. 4A). In comparison, lipoamides **4** and **5** caused weak swarming response at 200 μg (Fig. 4B). The purified amicoumacin B at a concentration of 100 μg/disk demonstrated inhibitory activity against *V. parahaemolyticus* PSU5429, while surfactin D (20 μg/disk) itself did not exhibit any antimicrobial activity. Intriguingly, when combined at lower concentrations, amicoumacin B (20 μg/disk) and surfactin-**3** (20 μg/disk) were able to restore the inhibitory activity observed at 100 μg/disk of amicoumacin B alone (Fig. 4C). We hypothesize that this may partly reflect a biosurfactant-mediated spreading artifact (altered surface tension and diffusion) or a possible synergistic effect between amicoumacin B and surfactins. Due to limited material, we were unable to distinguish between these possibilities. Future experiments using a nonionic surfactant (e.g., Tween 80) combined with amicoumacin B in the same disk-diffusion setup, along with a broth MIC checkerboard assay to calculate the fractional inhibitory concentration (FIC) index, may help distinguish between these possibilities. The motility assays were repeated against three other pathogenic marine *Vibrio* spp. of concern to aquaculture. Swarming motility was induced by surfactins in the oyster pathogen *V. coralliilyticus* RE22, but not for *V. harveyi* BB120 or the fish pathogen *V. anguillarum* NB10Sm (Fig. 4D). Neither *V. parahaemolyticus* PSU5429 nor *V. coralliilyticus* RE22 was observed to swarm in control experiments conducted in the absence of compounds from *B. pumilus* YP001.

## DISCUSSION

Decoding the ecological roles of microbial specialized metabolites is challenging due to the intricate nature of simultaneous responses to multiple signals and the small scale at which these interactions take place. Here, we used a combination of MALDI-TOF IMS, genome analysis, and bioactivity-guided isolation to uncover specialized metabolites produced by *B. pumilus* YP001 during its interaction with *V. parahaemolyticus* PSU5429. Four compounds were found to promote swarming motility in *V. parahaemolyticus*, including two surfactins (**2** and **3**) and the newly identified lipoamides B2 and C. In addition, the isocoumarin antibiotic amicoumacin B demonstrated antibiosis effects and contributed to a growth inhibition zone surrounding the *Bacillus* colony. The results from this study suggest that these relationships are mediated by complex chemical interactions, where multiple specialized metabolites work in concert to achieve advantageous outcomes. As *Bacillus* and *Vibrio* species co-occur in diverse marine environments and tend to compete for resources, including nutrients and space, understanding the roles of specific molecules and their combinations could provide valuable insights for influencing bacterial behavior in aquaculture systems and reducing infections.

In the observed scenario, the *Bacillus* colony appears to use a combination of specialized metabolites to create a “catch and kill” effect on *Vibrio* prey. The production of amicoumacins and surfactins, which are common within the genus, may serve as a strategy to obtain nutrients from nearby cells. However, further studies are needed to explore the *Bacillus* colony’s ability to utilize nutrients released by lysed vibrios. One potential approach is investigating the use of radio-labeled vibrios to track their uptake and assimilation by the *Bacillus* colony. Alternatively, the *Bacillus* might produce amicoumacin B as a defense mechanism against the approaching *Vibrio*. Not all *Vibrio* species exhibit swarming behavior, which suggests strain variability or an adaptive defense developed by *Bacillus* to counter this specific tactic. Amicoumacins also show promise as antibiotics for combating infection outbreaks in aquaculture facilities. For example, they have demonstrated efficacy in targeting *V. harveyi* infections in shrimp and *V. anguillarum* infections in fish (32). These antibiotics offer a unique mechanism of action that may not confer cross-resistance with clinically used antibiotics, making them potentially valuable in treating antibiotic-resistant infections.

This example of bacterial co-culture experiments, combined with IMS, genome sequencing, and chemical analyses, illustrates a valuable approach for exploring the functions of specialized metabolites in chemically mediated interactions. Co-culturing provides an opportunity to uncover complex interactions driven by various molecules that affect microbial neighbors. The concurrent secretion of specialized metabolites in response to nearby pathogens may offer new insights into drug combinations that effectively mitigate infections. As observed with *B. pumilus* YP001, a combination of compounds that simultaneously affected motility (surfactins) and survival (amicoumacins) created a lethal outcome for members of the *V. parahaemolyticus* colony. The future combination of these experimental techniques will undoubtedly contribute to a better understanding of the ecological roles and functions of different microbial species within a community context and further reveal strategies employed in nature to influence bacterial behavior and survival.

## MATERIALS AND METHODS

### General experimental procedures

NMR experiments were conducted using an Agilent Inova NMR 500 MHz spectrometer in DMSO-*d*_6_ (99.99%, Sigma-Aldrich, St. Louis, MO, USA) at 25°C (referenced to residual DMSO at *δ*_H_ 2.54 and *δ*_C_ 39.5). High-resolution electrospray ionization mass spectra (HR-ESI-MS) and tandem mass spectra were acquired using a TripleTOF 4600 spectrometer (AB Sciex) operating in negative ion mode. Flash chromatography was completed using an Agilent 971-FP flash purification system (Agilent Technologies, Santa Clara, CA, USA) with Biotage SNAP KP-C18-HS 120g cartridges (Biotage, Charlotte, NC, USA). HPLC experiments were performed on a Shimadzu Prominence-I LC-2030C equipped with a PDA detector model LC-2030/2040, pump LC-2030, and autosampler LC-2030. Optical rotations were measured on a Jasco P-2000 polarimeter, and UV spectra were taken on a Beckman Coulter DU 800. IR experiments were carried out on Thermo Nicolet 380 FT-IR. MALDI-TOF IMS was carried out on a Bruker Autoflex Speed LRF mass spectrometer.

### Isolation, sequencing, and computational genome analysis of *Bacillus pumilus* YP001

Strain YP001 was isolated from the egg mass of a female American lobster (*Homarus americanus*) collected in Narragansett Bay (12/16/15; Fort Weatherall, Jamestown, RI, USA). The eggs were gently brushed with a sterile cotton swab, which was then stored in a sterile sampling bag for transportation to the lab. The cotton tip was transferred into 1.5 mL of sterile artificial seawater (Instant Ocean; Spectrum Brands) under aseptic conditions and then vortexed. A 100 μL aliquot was plated on artificial seawater agar plates supplemented with yeast (1 g/L) and peptone (5 g/L) (YP30). YP001 was picked from the plate as a single colony, streaked to purity (twice on YP30 plates), and cryogenically stored at −80°C in glycerol stocks.

Total DNA was extracted from overnight cultures of *Bacillus pumilus* YP001 grown in liquid YP30 using the Promega Wizard Genomic DNA Purification Kit (Promega, USA). The DNA was resuspended in 2 mM Tris-HCl buffer (Bio Basic). DNA was quantified using a NanoDrop 1000 spectrophotometer (ND-1000) and checked for quality on a 1% agarose gel stained with ethidium bromide. The resulting DNA was sequenced on an Illumina MiSeq sequencer at the Genomics and Sequencing Center, University of Rhode Island. Total genomic DNA was sheared by sonication (Covaris S220), and paired-end libraries were prepared using the SMARTer PrepX DNA Library Kit on a SMARTer Apollo system (Takara Bio, USA). Reads were trimmed using the CLC Genomic Workbench (version 9.5.3, Qiagen, Hilden, Germany) for quality, ambiguous base pairs, adaptors, duplicates, and size with default parameters. A draft genome was assembled using the SPAdes assembler (Algorithmic Biology Lab, St. Petersburg Academic University of the Russian Academy of Sciences). Coverage was determined by mapping reads to a draft genome of *Bacillus pumilus* (CP016784.1). BGCs are annotated by antiSMASH v8 (cite: antiSMASH 8.0: extended gene cluster detection capabilities and analyses of chemistry, enzymology, and regulation). The BGC of surfactin in *B. velezensis FZB42* (BGC0000433) and BGC of lichenysin in *B. licheniformis* DSM 13 = ATCC 14580 (BGC0000381) are retrieved from MIBiG v4 (33). Gene cluster alignments of BGCs are performed by clinker, and only the best matches are displayed. The draft genome assembly has been deposited in DDBJ/EMBL/GenBank under accession number PRJNA434728. Sequence alignments for amicoumacin biosynthetic cassettes were analyzed for identity using Geneious and NCBI Delta-BLASTp.

### Isolation and structure elucidation of YP001 specialized metabolites

*B. pumilus* YP001 was grown in 9 × 1 L of YP30 broth for 7 days at 25°C and 150 rpm on a New Brunswick C10 rotary shaker table in the presence of Amberlite XAD-16 resin (10 g/L; Sigma Aldrich). Following cultivation, the resin was collected by filtering through two layers of cheesecloth, washed three times with DI water (4 L total) to remove salts, and then extracted with 500 mL of 1:1 CH_2_Cl_2_ and CH_3_OH, followed by 1 L of CH_3_OH, and finally 200 mL of EtOAc. The combined solvents were removed by rotary evaporation, and the resulting extract was partitioned between 100 mL H_2_O and three washes of 500 mL EtOAc. The combined organic layers were concentrated *in vacuo* to yield 0.8 g of extract. The organic fraction was separated by flash chromatography using a Combiflash Rf200 equipped with a 50 g C18 RediSep Rf High-Performance Gold column (Teledyne ISCO). A linear solvent gradient of 5–100% CH_3_OH in H_2_O (0.1% formic acid) was run over 20 min at a flow rate of 40 mL/min, and then the mobile phase was held at 100% CH_3_OH for 5 min. Individual peaks were collected by monitoring UV absorbance at 254 nm, yielding six fractions (QW1-144-1 to QW1-144-6). HPLC separations were completed with a Waters XBridge preparative C18 5 μm, 10 × 250 mm column at 4 mL/min, using a linear solvent gradient of 5–100% B (MeOH) over 16 min against solvent A (water + 0.1% formic acid). Solvent B was held at 5% for 4 min prior to starting the gradient, and 100% B was held for a subsequent 4 min following the gradient. Pure lipoamide B2 and C were isolated by preparative HPLC following the same solvent gradient, yielding 11.8 mg and 11.9 mg, respectively. Analytical HPLC experiments were carried out on a Waters XBridge Analytical C18 5 μm, 4.6 × 250 mm column at 1 mL/min, using the same mobile phase methods. Purification of surfactin C15 and D followed a solvent gradient of 80–100% B (MeOH) run over 16 min against solvent A (water + 0.1% formic acid). Solvent B was held at 80% for 4 min prior to the gradient beginning, and 100% B was held for a subsequent 4 min. Amicoumacin B was isolated to purity through flash chromatography collection. In total, amicoumacin B, surfactin-**2**, and surfactin-**3** were obtained in yields of 11.6 mg, 2.6 mg, and 6.3 mg, respectively.

### Antimicrobial activity assays

B. pumilus YP001 was tested for antimicrobial activity against the pathogens *Vibrio anguillarum* NB10Sm, *Vibrio harveyi* BB120, *Vibrio coralliilyticus* RE22, and *Vibrio parahaemolyticus* PSU5429 through a modified diffusion assay. *V. parahaemolyticus* PSU5429 was grown for 24 h in YP30, diluted 10^−3^-fold, and 100 μL of the diluted culture was spread on 15 mL of YP30 agar. A 5 μL spot of *B. pumilus* YP001, grown overnight in 10 mL YP30, was added in triplicate and incubated at 25°C. Zones of inhibition were imaged with a Bio-Rad Gel Doc Universal Hood after 24–48 h at 25°C.

### Swarming motility assay

*B. pumilus* YP001 colonies, extracts, and purified compounds were tested for induction of swarming motility in *V. parahaemolyticus* PSU5429. Sterile disks were loaded with 20 or 100 μg of test compounds and placed onto the surface of a YP30 agar plate. Alternatively, a 5 μL spot of YP001 grown for 24 h in 5 mL of YP001 broth was spotted onto a 15 mL YP30 agar plate. PSU5429 was grown for 24 h in YP30 broth, diluted to 1 × 10^5^ CFU/mL in sterile media, and spotted at a distance of 3, 8, or 13 mm from the disk or colony. Plates were incubated for 24 h at 27°C and then imaged with a Bio-Rad Gel Doc Universal Hood. This assay is qualitative for the induction of swarming motility.

### MALDI-TOF IMS imaging

MALDI-TOF IMS experiments were carried out on thin YP30 plates (10 mL in 90 mm Petri dishes). Strains were plated in triplicate and incubated for up to 96 h. MALDI-TOF MSI was carried out on a Bruker Autoflex Speed LRF mass spectrometer. These colonies were wet-mounted onto a stainless steel 96-spot MALDI target plate, coated with a 50:50 *α*-cyano-hydroxycinnamic acid (CHCA) and dihydroxybenzoic acid (DHB) matrix using a 53 μm strain steel sieve, dried in a desiccator for 4 h at 37°C, and analyzed by MALDI-TOF MSI. Pictures of the sample were taken wet, after matrix application, and after desiccation for each point in the software. Samples were analyzed by MALDI-TOF MSI at 80% laser power, 10.5× gain, and 500 μm raster in positive ion reflectron mode on a Bruker Autoflex Speed LRF mass spectrometer. Data were normalized using root mean square (26) as indicated, using FlexImaging v. 5.0.

## SUPPLEMENTAL MATERIAL

Supplemental File msystems.01440-25-s0001.pdf Table S1; Figures S1-S30.

ASM does not own the copyrights to Supplemental Material that may be linked to, or accessed through, an article. The authors have granted ASM a non-exclusive, world-wide license to publish the Supplemental Material files. Please contact the corresponding author directly for reuse.
