# Supplementary material for: Lipopeptides and antibiotics from a marine Bacillus pumilus mediate a potential “catch and kill” effect on pathogenetic Vibrio parahaemolyticus
Source: mSystems. 2025 Dec 31;11(2):e01440-25. doi: 10.1128/msystems.01440-25 (PMC12911350; doi:10.1128/msystems.01440-25)

Lipopeptides and antibiotics from a marine *Bacillus pumilus* mediate a potential 'catch and kill' effect on pathogenetic *Vibrio parahaemolyticus*

Hilary J. Ranson,[1,#] Yan-Song Ye,[2] Valentina Z. Petukhova,[3] Abigail Green-Saxena,[3] Ruolin He,[4] Jiadong Sun,[1] Bhaskar Godugu,[5] Laura M. Sanchez,[3,6] Qihao Wu,[2,*] David C. Rowley[1,*]

[1]Department of Biomedical & Pharmaceutical Sciences, The University of Rhode Island Kingston, RI 02881, United States
[2]Department of Pharmaceutical Sciences, University of Pittsburgh, Pittsburgh, PA 15261, United States
[3]Department of Pharmaceutical Sciences, University of Illinois Chicago, Chicago, IL, 60612, United States
[4]Bioinformatics Group, Wageningen University, Droevendaalsesteeg 1, 6708 PB Wageningen, Netherlands.
[5]Department of Chemistry, University of Pittsburgh, Pittsburgh, PA 15261, United States
[6]Department of Chemistry and Biochemistry, University of California Santa Cruz, Santa Cruz, CA, 95064, United States

*Corresponding authors
Qihao Wu, Email: qiw153@pitt.edu
David C. Rowley, Email: drowley@uri.edu

Table S1. ^{1}H and ^{13}C NMR data (δ in ppm, J in Hz) for compound **1** in DMSO-d_6. Key ^{1}H-^{1}H COSY and HMBC correlations of compound **1** are are shown.

Position	Type	δ_C	δ_H
1	C	169.0	
3	CH	81.0	4.69 dt (12.6, 2.8)
4	CH_2	29.0	3.04 dd (16.9, 12.8)
			2.86 dd (16.9, 2.5)
5	CH	118.5	6.81 d (7.4)
6	CH	136.2	7.48 t (8.0)
7	CH	115.2	6.85 d (8.3)
8	C	160.9	
9	C	108.3	
10	C	140.7	
1'	CH_3	23.3	0.90 d (6.4)
2'	CH_3	21.5	0.85 d (6.4)
3'	CH	24.0	1.67, overlap
4'	CH_2	40.0	1.67, overlap
			1.33, m
5'	CH	48.1	4.20, m
	NH		7,91, d (9.3)
7'	C	172.6	
8'	CH	71.6	3.96 d (7.5)
9'	CH	71.8	3.72, m
10'	CH	50.1	3.31, m
11'	CH_2	33.4	2.32, d (16.9)
			2.19 dd (16.9, 10.2)
12'	C	173.7	

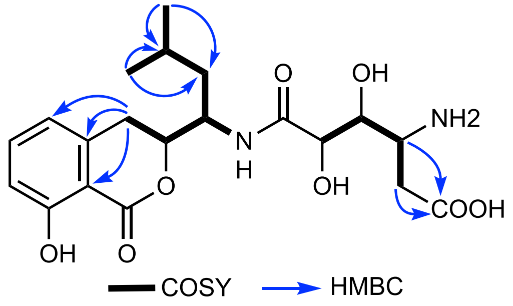

Figure S1. *Bacillus pumilus* YP001 colonies produce clear zones of inhibition on lawns of tested *Vibrio* pathogens. Zones of inhibition produced by *Bacillus pumilus* YP001 against *Vibrio harveyi* BB120 (top), *V. anguillarum* NB10Sm (middle), and *V. coralliilyticus* RE22 (bottom) after 24 h on YP30 agar at 27 °C.

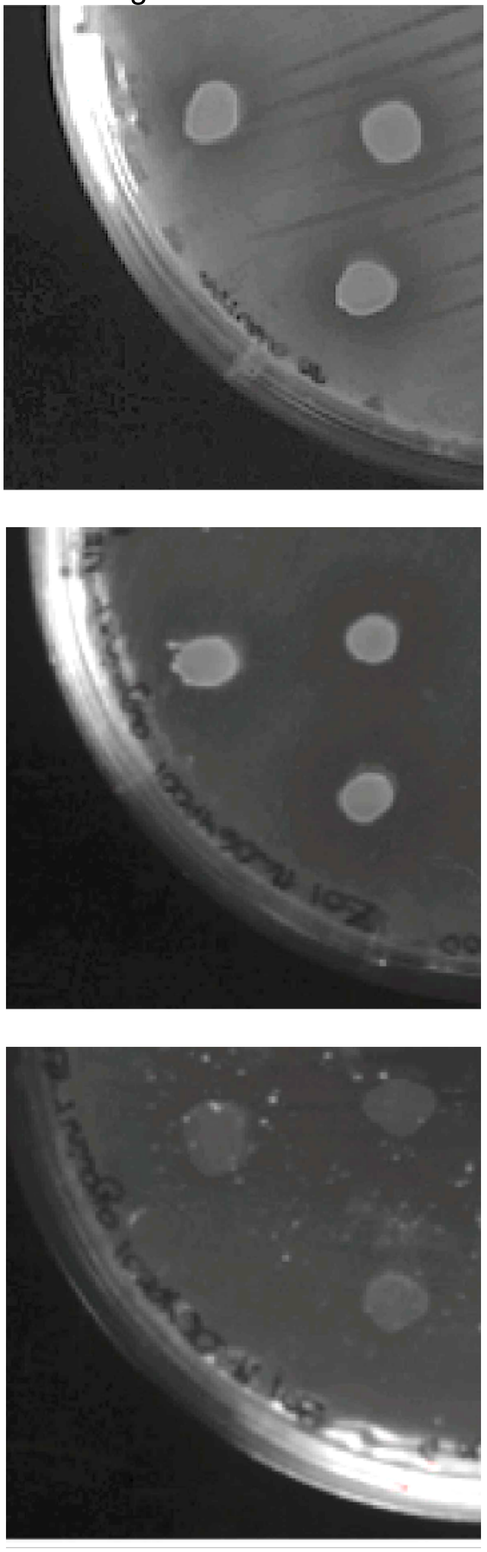

Figure S2. TIC for the combined spectrum with RMS normalization. The trace represents the sum of all ions detected across all pixels over the full *m/z* range, illustrating the overall ion distribution and relative signal intensities. Vertical lines mark the ions at *m/z* 425.2 $[M+H]^+$, 1058.7 $[M+Na]^+$, and 1072.7 $[M+Na]^+$ that were used for imaging.

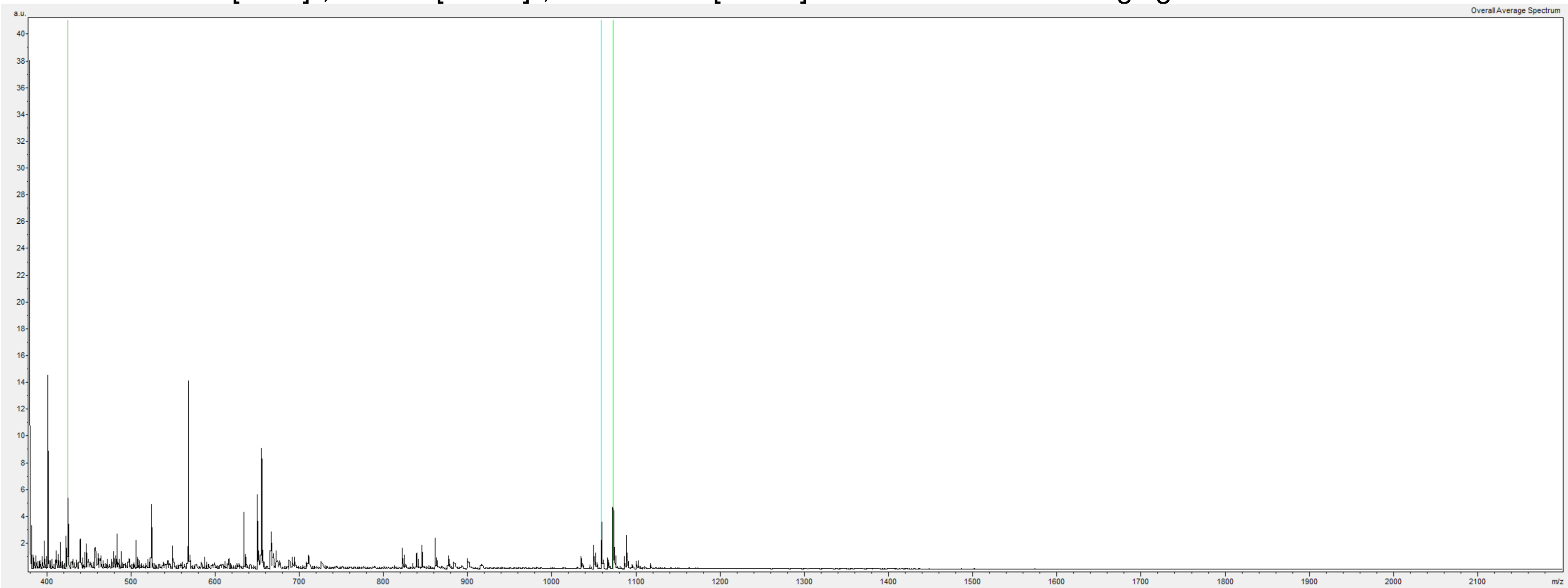

Figure S3. Spectra overlay for surfactin range. Highlighted in grey is the mass filter mark for *m/z* 1058.7 +/- 0.25 Da. Highlighted in green is the mass filter mark for *m/z* 1072.7 +/- 0.25 Da. Black is the combined average spectrum, dark blue is the interaction spectrum, red is the control amicoumacin extract spotted on agar, light blue is the blank agar media.

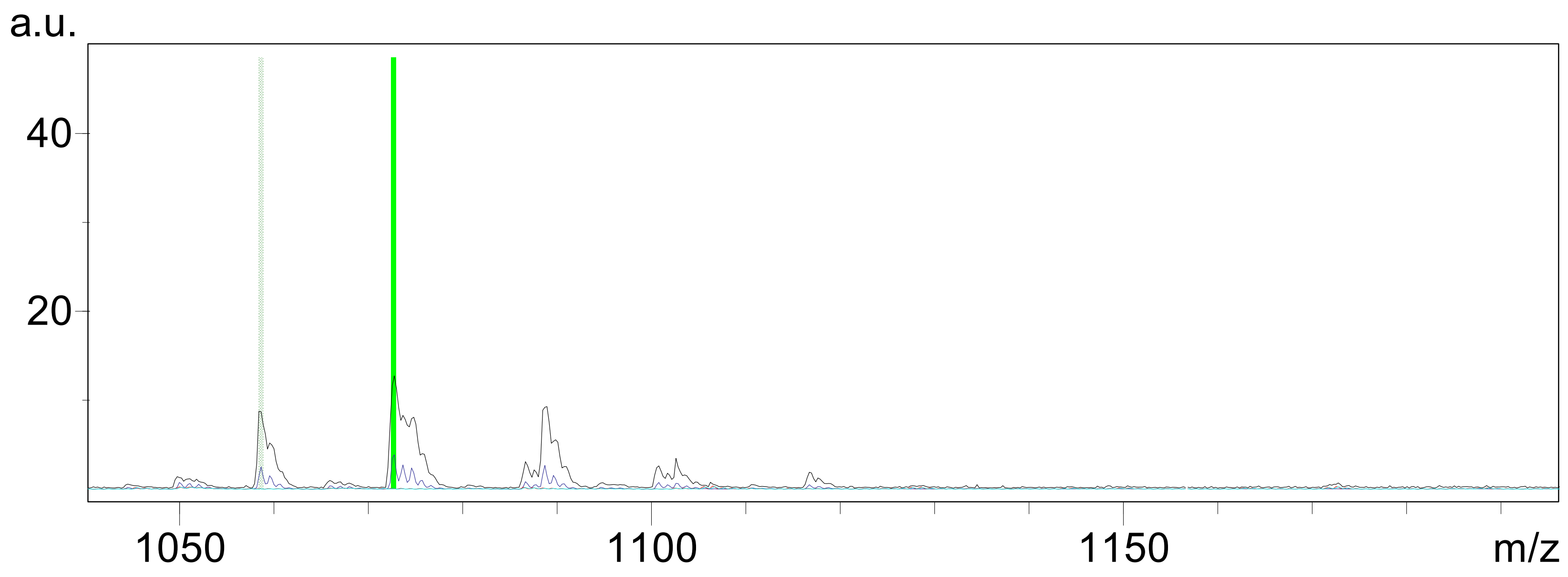

Figure S4. Spectra overlay for amicoumacin range. Highlighted in grey is the mass filter mark for *m/z* 425.2 +/- 0.25 Da. Black is the combined average spectrum, dark blue is the interaction spectrum, red is the control amicoumacin extract spotted on agar, light blue is the blank agar media.

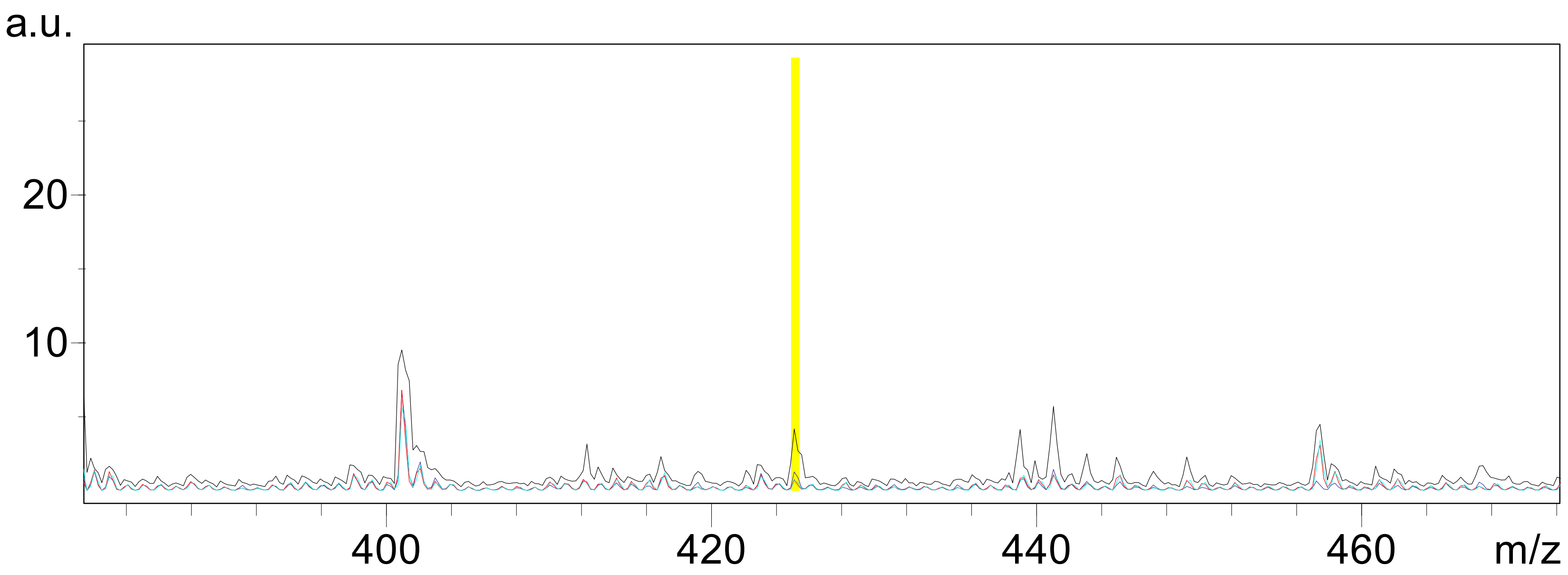

Figure S5. Comparative analysis of lipopeptide NRPS gene clusters and A-domain specificities. Domain organizations of the canonical lichenysin cluster (*Bacillus licheniformis* ATCC14580), surfactin clusters (*B. velezensis* FZB42, *B. pumilus* NMSW10), and the putative lipopeptide cluster in *B. pumilus* YP001 (region 3.1) are shown. Predicted A-domain substrate specificities indicate that the A1 domain of module 1 in YP001 activates Glu, matching surfactins and contrasting with lichenysin, which incorporates Gln at this position. Notably, genes encoding the NRPS modules that install the Val-Asp-D-Leu motif in surfactin (e.g., *srfAB* in FZB42 and *C6X96_RS144990* in NMSW10) are absent from YP001. Red dashed boxes denote assembly gaps. C, condensation; A, adenylation; CP, carrier protein; E, epimerase; TE, thioesterase; CAL, CoA-ligase-like starter; KR, ketoreductase. Arrows represent ORFs.

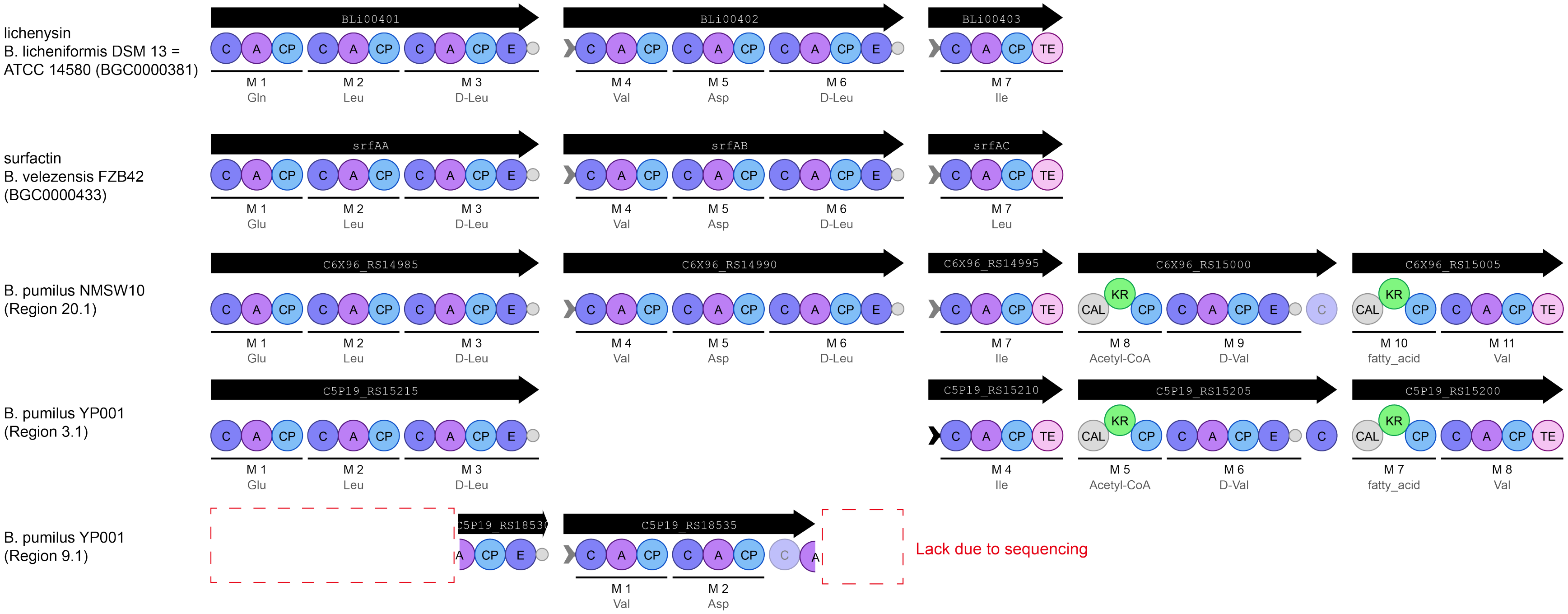

Figure S6. Comparison of lipopeptide BGCs reveals a surfactin-like NRPS fragment in *Bacillus pumilus* YP001. (Top) Comparison of lipopeptide BGCs from *B. pumilus* YP001 (regions 3.1 and 9.1) with representative surfactin (*B. velezensis* FZB42 and *B. pumilus* NMSW10) and lichenysin (*B. licheniformis* DSM 13) clusters. Shaded ribbons indicate pairwise nucleotide identity (0 - 100%). A genome-wide search identified region 9.1 in YP001, which shares 73% sequence identity with the *B. pumilus* NMSW10 gene *C6X96_RS144990*, a *srfAB*-like NRPS subunit that installs the Val-Asp-D-Leu motif in surfactin. (Bottom) Direct alignment of YP001 region 9.1 against surfactin/lichenysin loci from the indicated *Bacillus* strains highlights its strongest similarity to the NMSW10 *srfAB*-like segment and its absence from YP001 region 3.1, consistent with region 9.1 representing the missing assembly piece for the Val-Asp-D-Leu module.

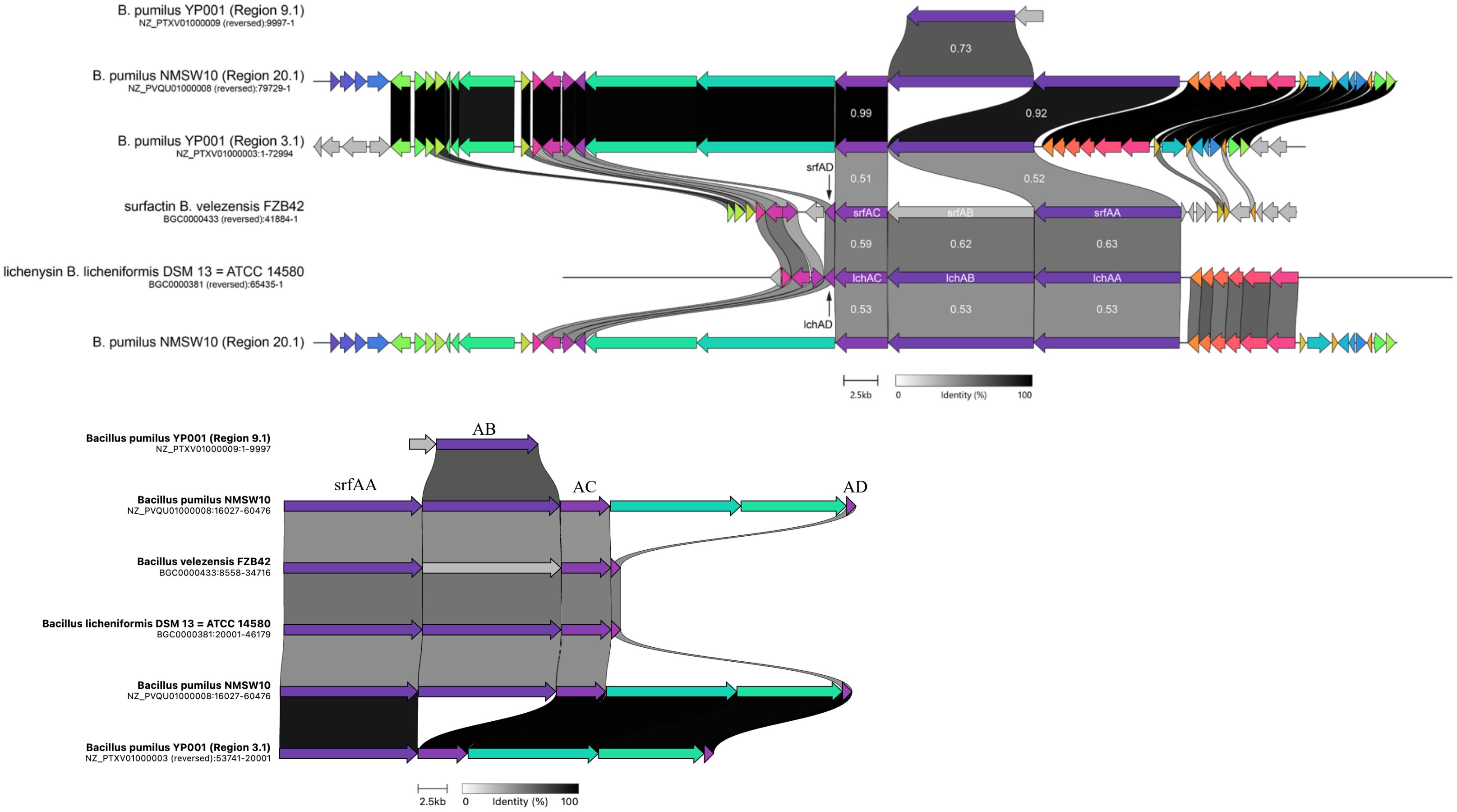

Figure S7. Comparison of the amicoumacin BGC in *B. pumilus* YP001 and related *Bacillus* strains. Amicoumacin locus from YP001 aligned to homologous regions in *B. inaquosorum* KCTC, *B. pumilus* SF214, *B. pumilus* SH-B9, and *B. subtilis* fmb60. Gene arrows denote ORFs and orientation; shaded ribbons indicate pairwise nucleotide identity (0 - 100%; scale bar, 2.5 kb). The core amicoumacin BGC is highly conserved and collinear across strains, with only minor strain-specific insertions/deletions in flanking segments, supporting assignment of the YP001 locus as an amicoumacin pathway.

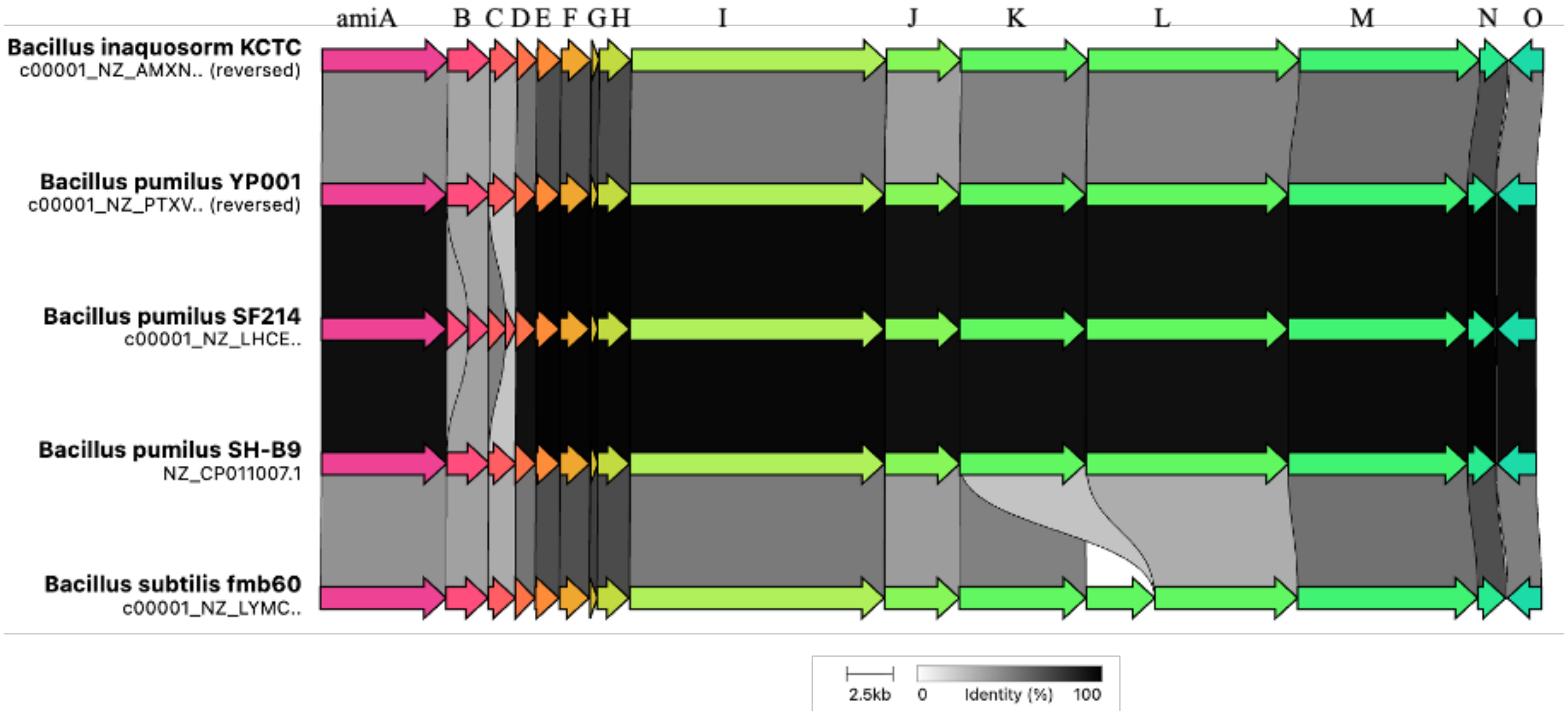

Figure S8. ^{1}H NMR spectrum of compound **1** in DMSO-d_6.

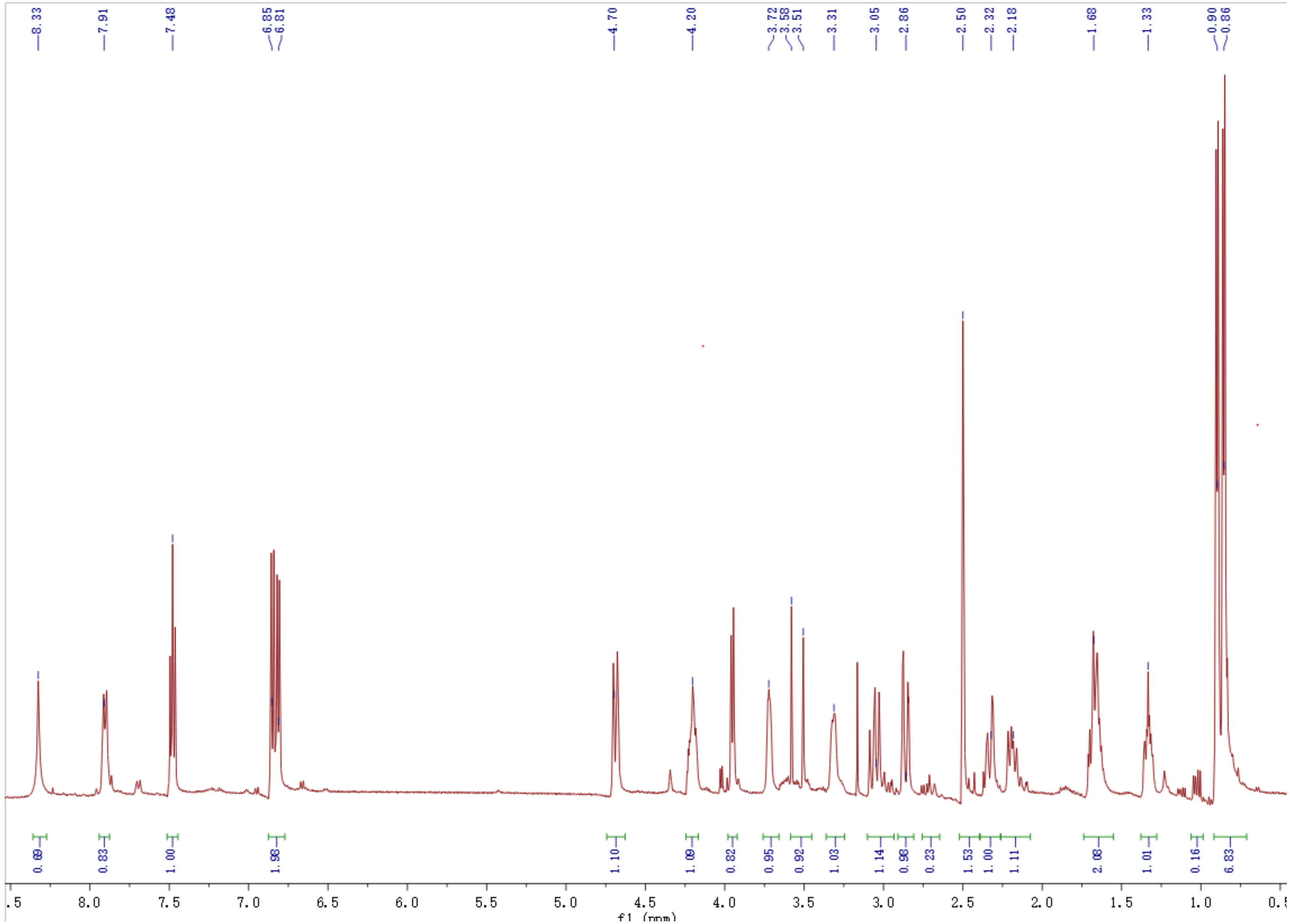

Figure S9. ^{13}C NMR spectrum of compound **1** in DMSO-d_6.

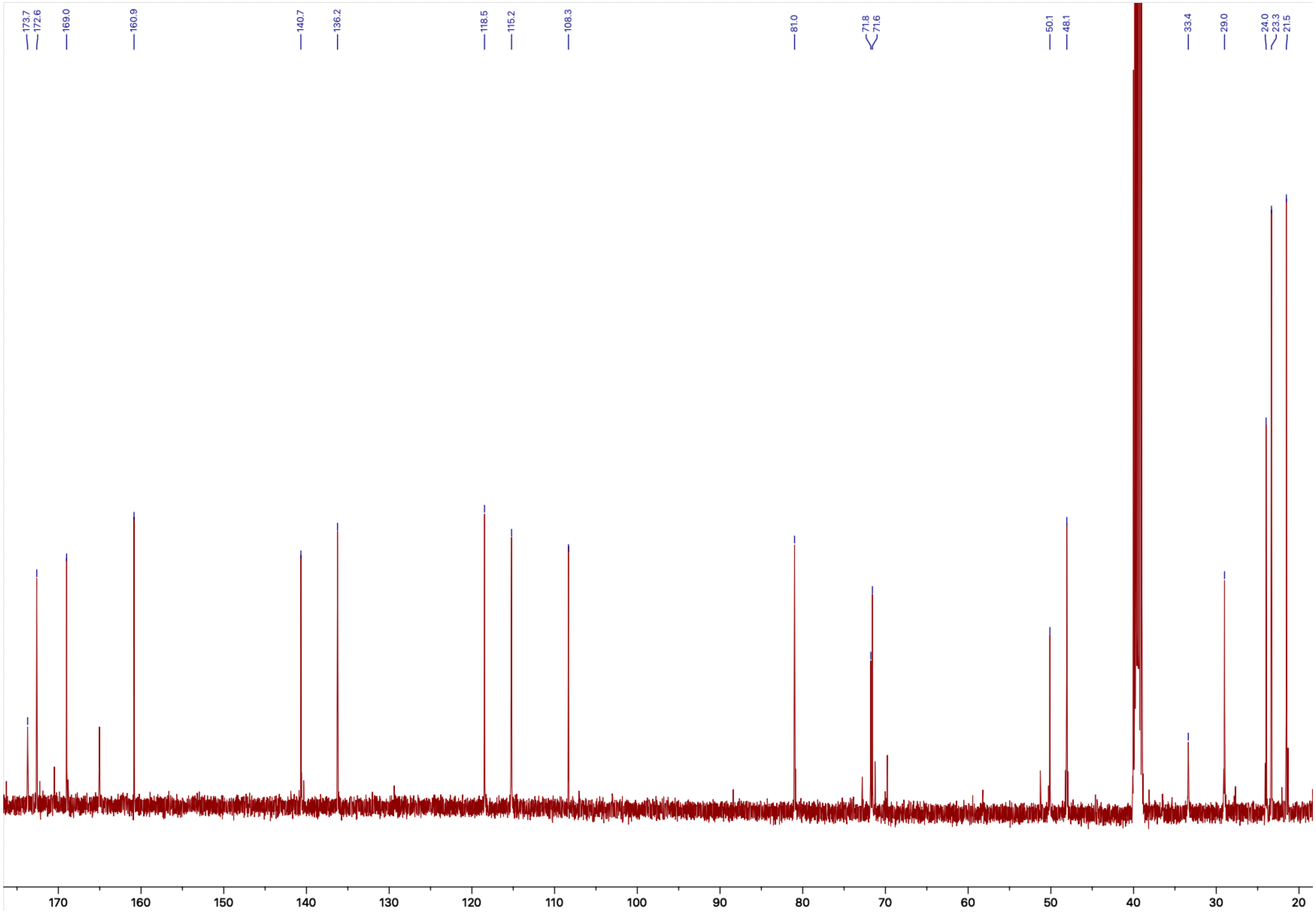

Figure S10. ^{1}H-^{1}H COSY spectrum of compound **1** in DMSO-d_6.

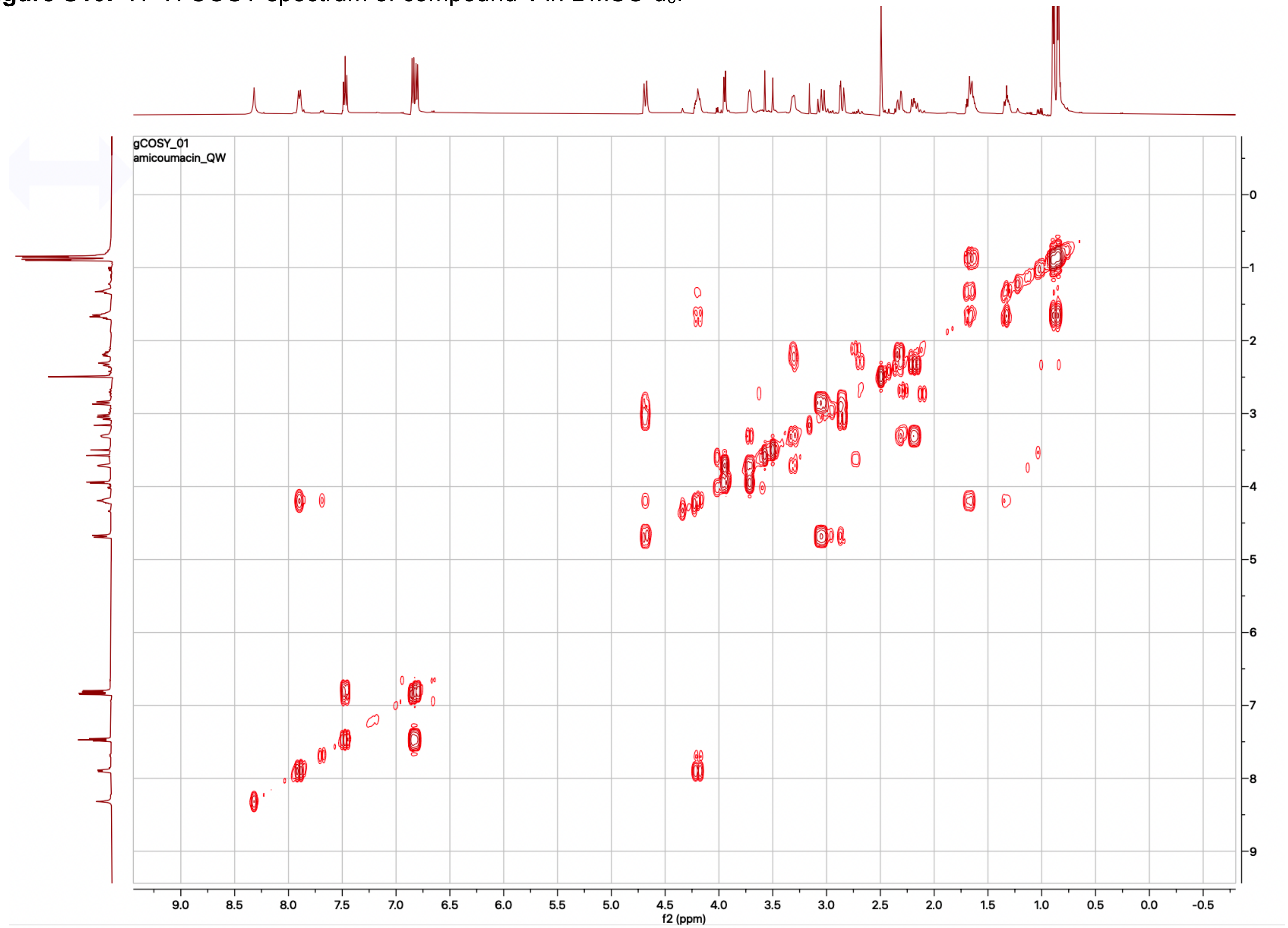

Figure S11. HSQC spectrum of compound **1** in DMSO-d_6.

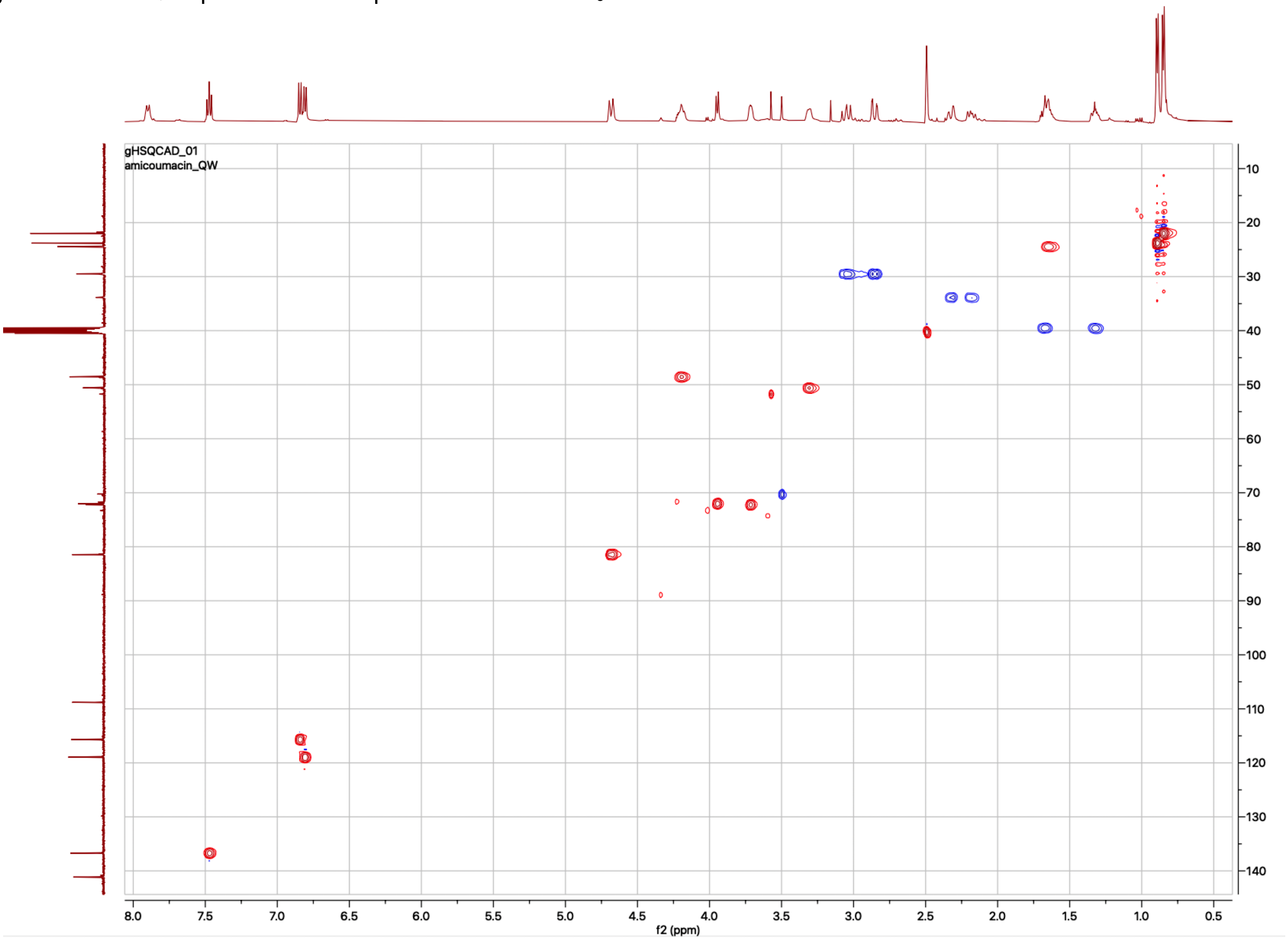

Figure S12. HMBC spectrum of compound **1** in DMSO-d_6.

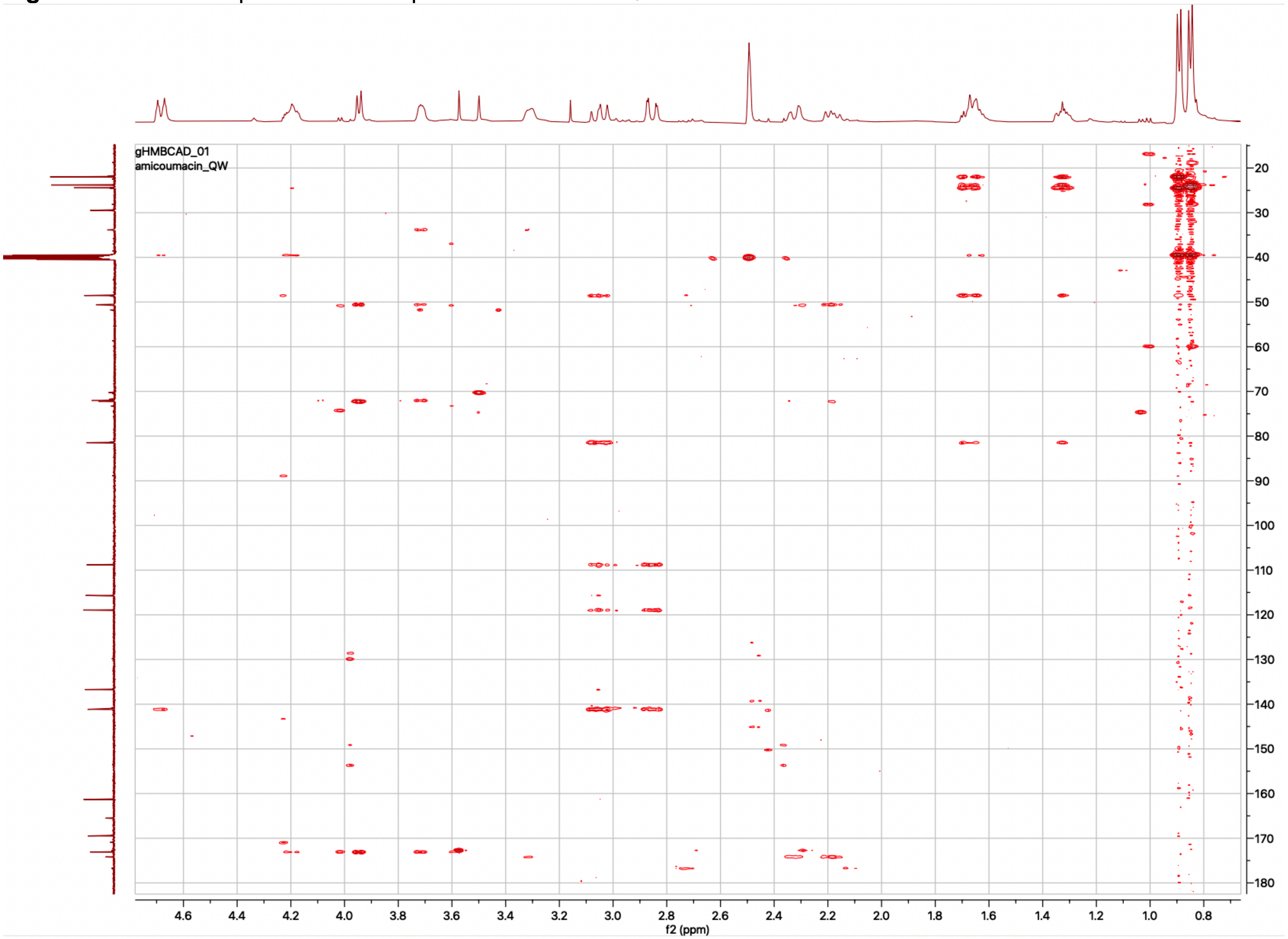

Figure S13. HR-ESI-MS of compound **1** in positive-ion mode.

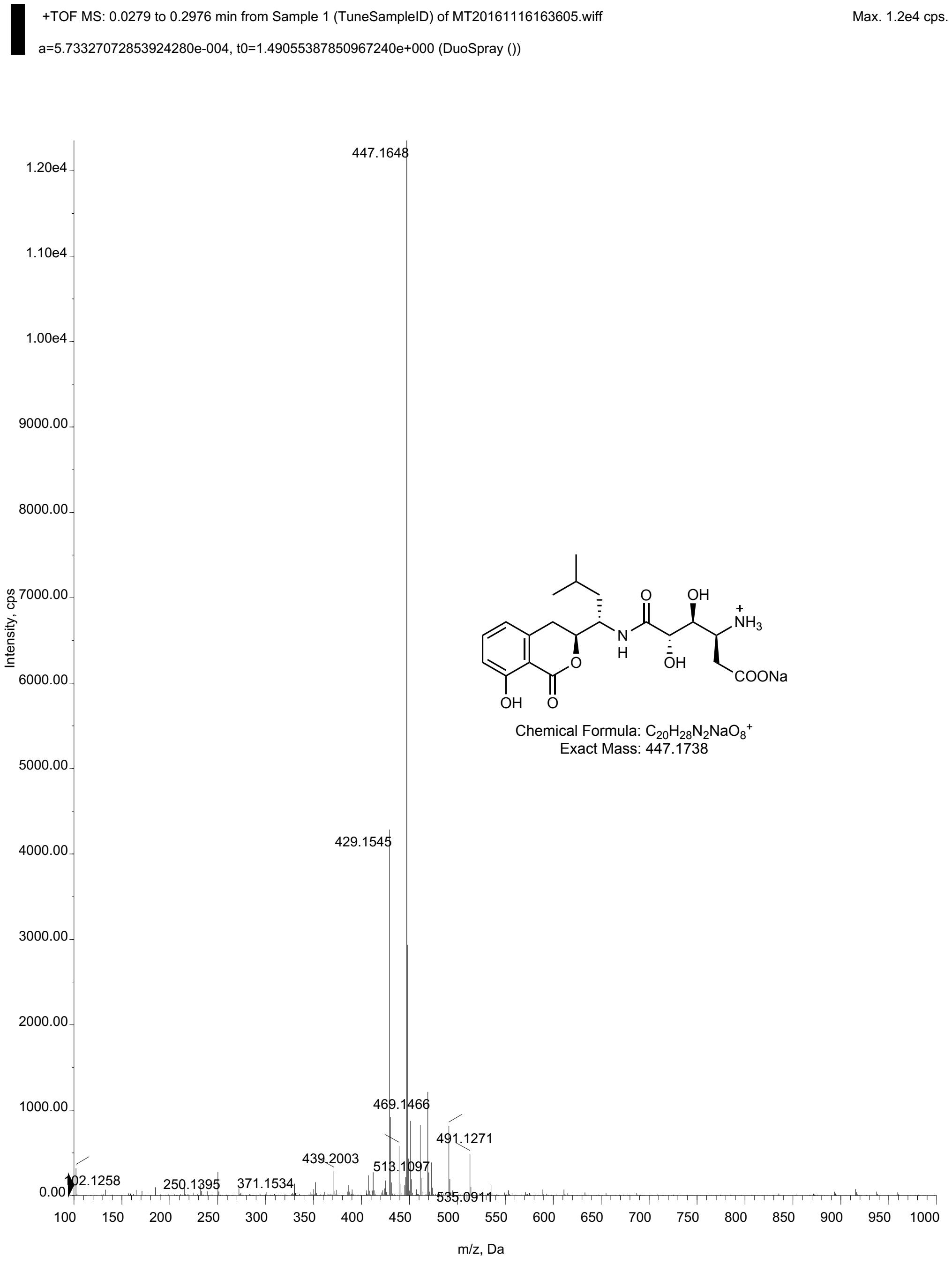

Figure S14. HR-ESI-MS of compound **2** in negative-ion mode.

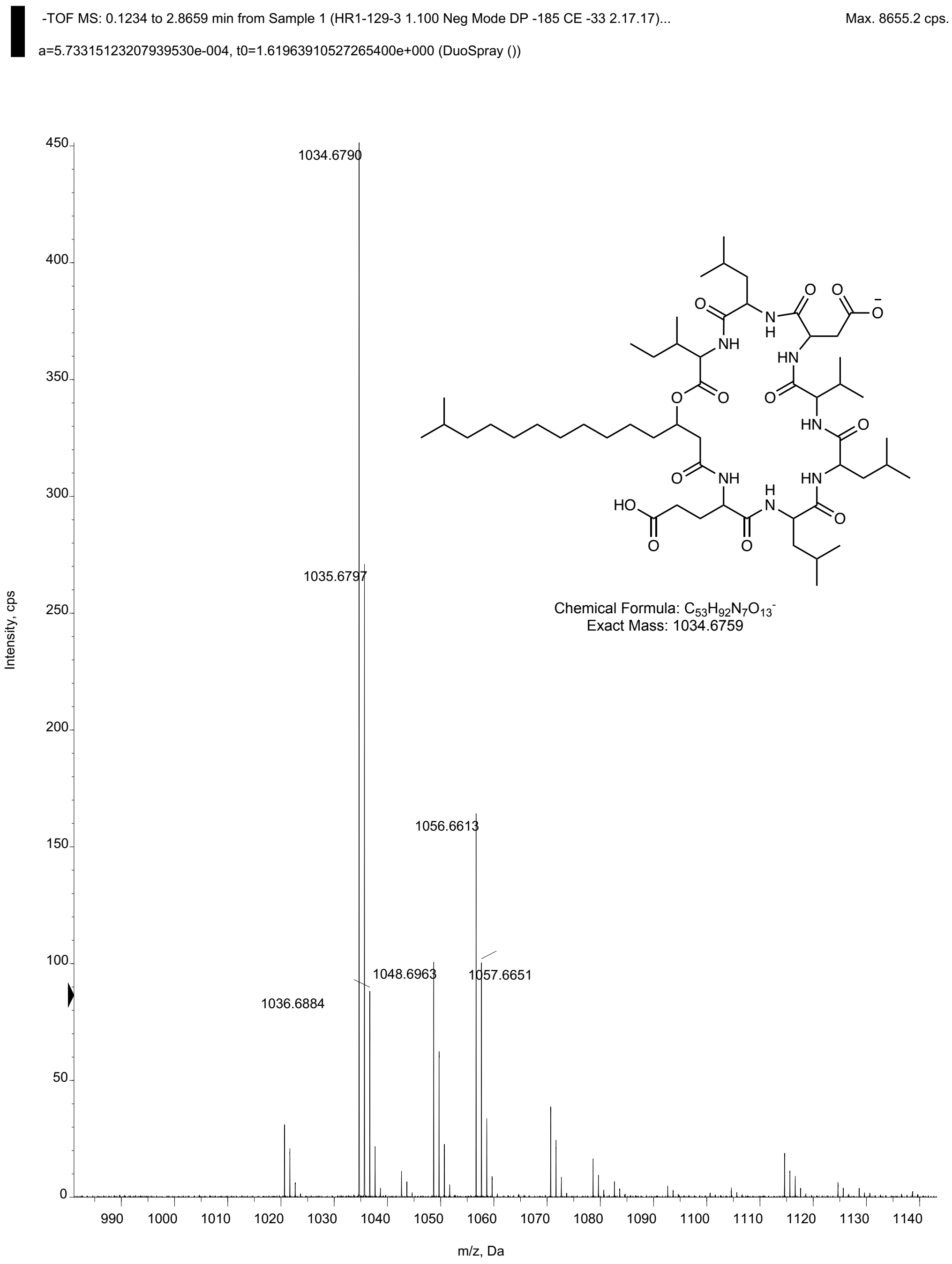

Figure S15. HR-ESI-MS of compound **2** in positive-ion mode.

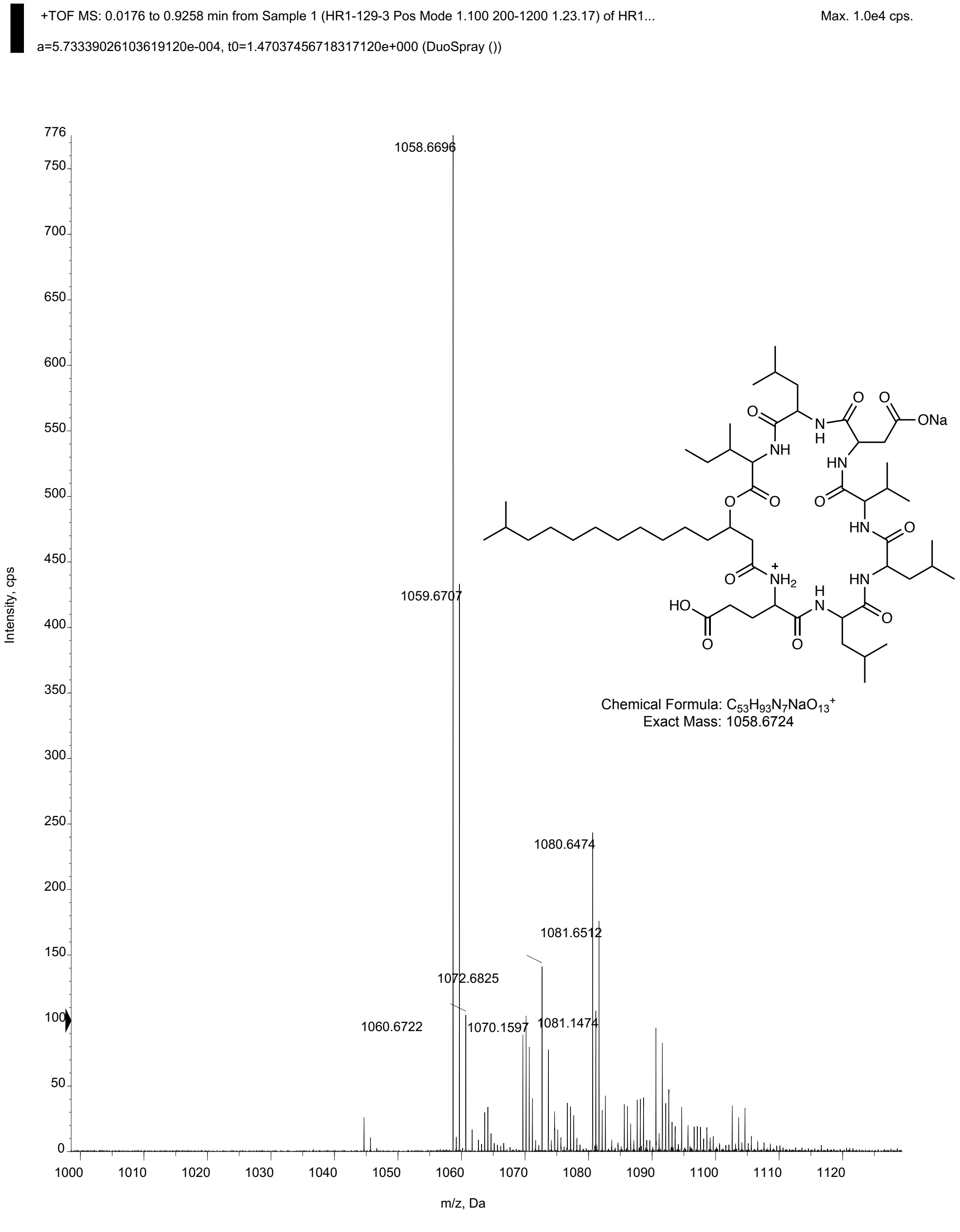

Figure S16. HR-ESI-MS of compound **3** in positive-ion mode.

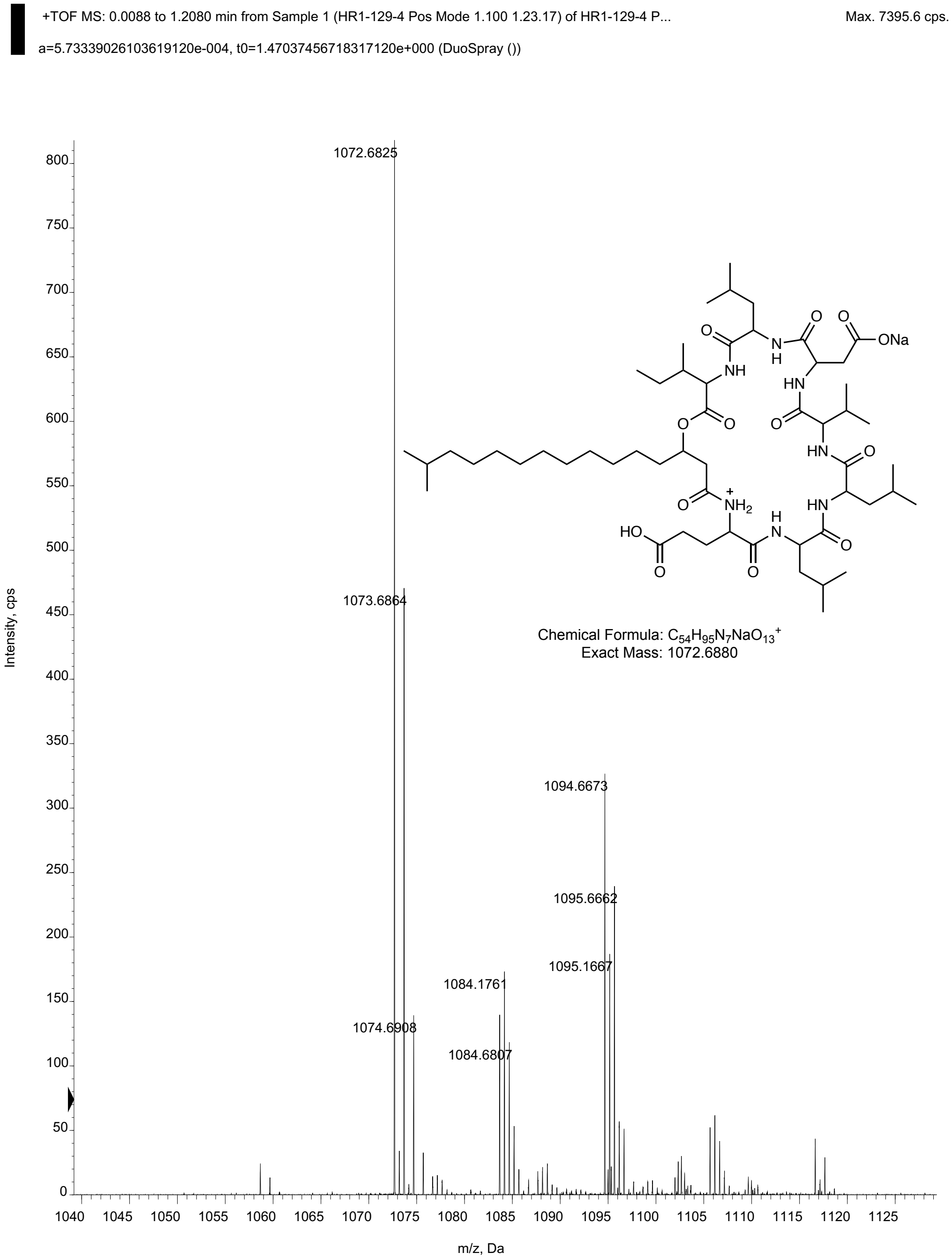

Figure S17. HR-ESI-MS of compound **3** in negative-ion mode.

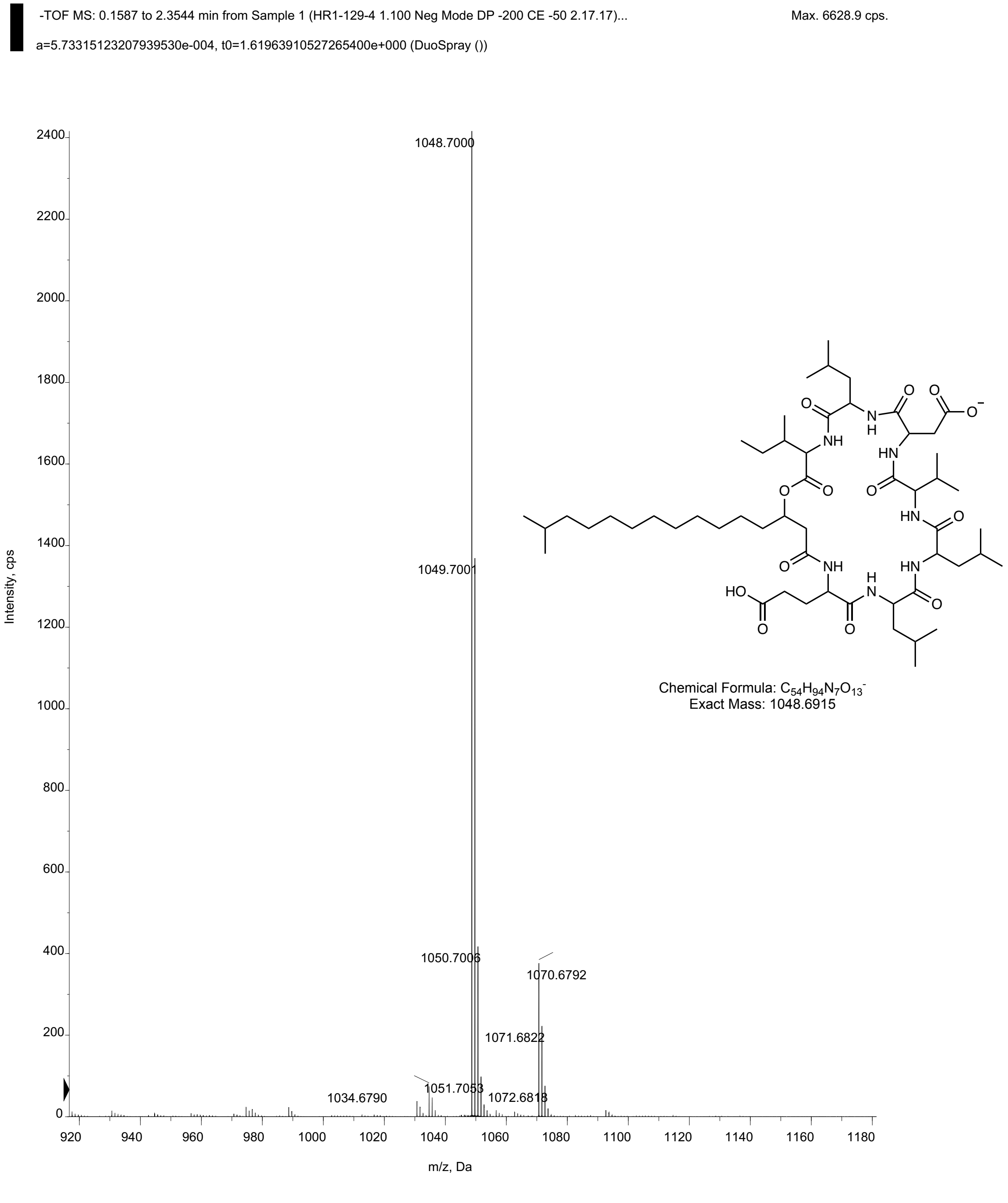

Figure S18. HR-ESI-MS of compound **4** in positive-ion mode.

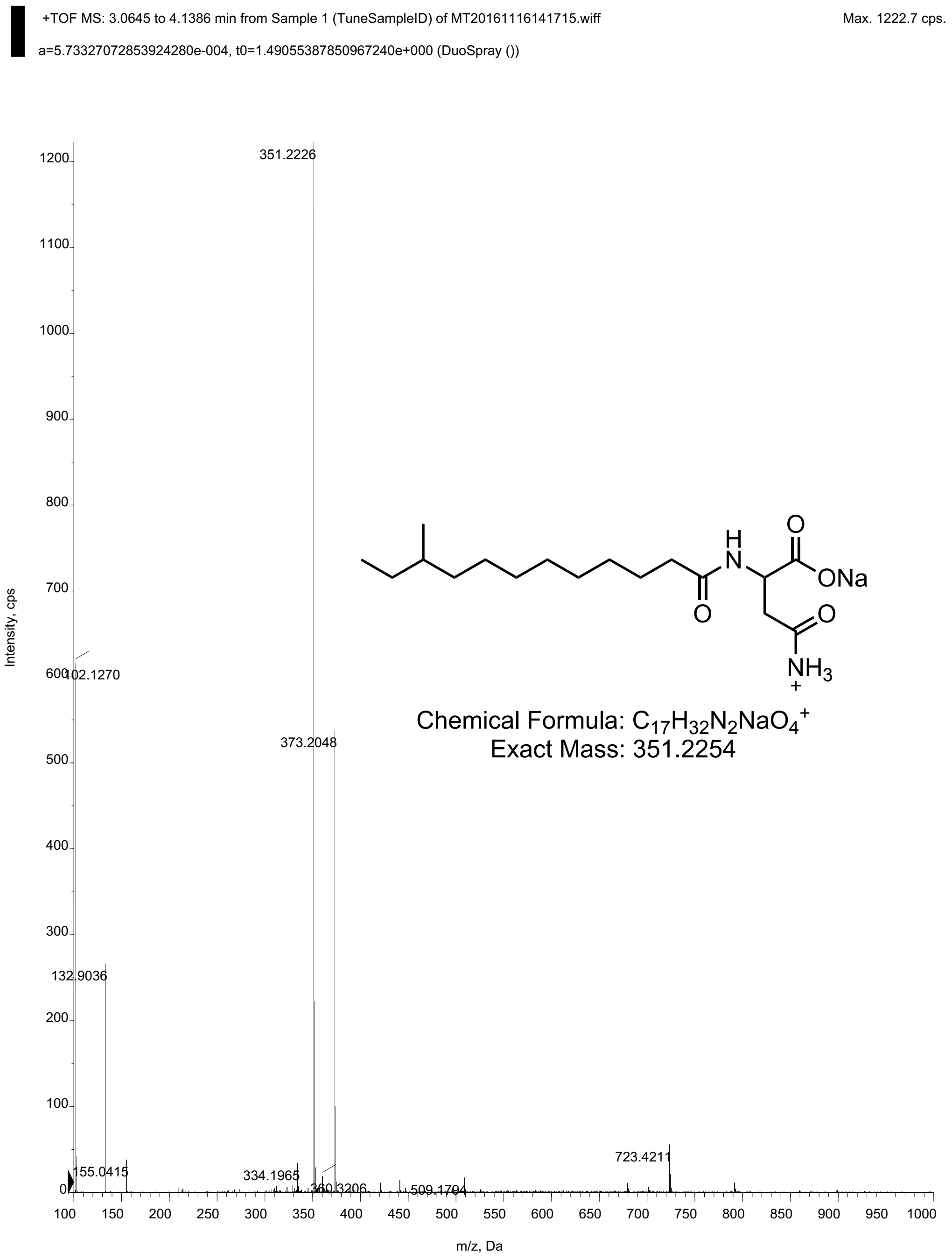

Figure S19. HR-ESI-MS/MS of compound **4** in positive-ion mode. Product-ion spectrum of $[M+H]^+$ with a key fragment labeled (fragment **a**). The diagnostic ion **a** at *m/z* 155.0393 is consistent with an Asn-derived fragment, confirming the presence of an asparagine unit in compound **4**.

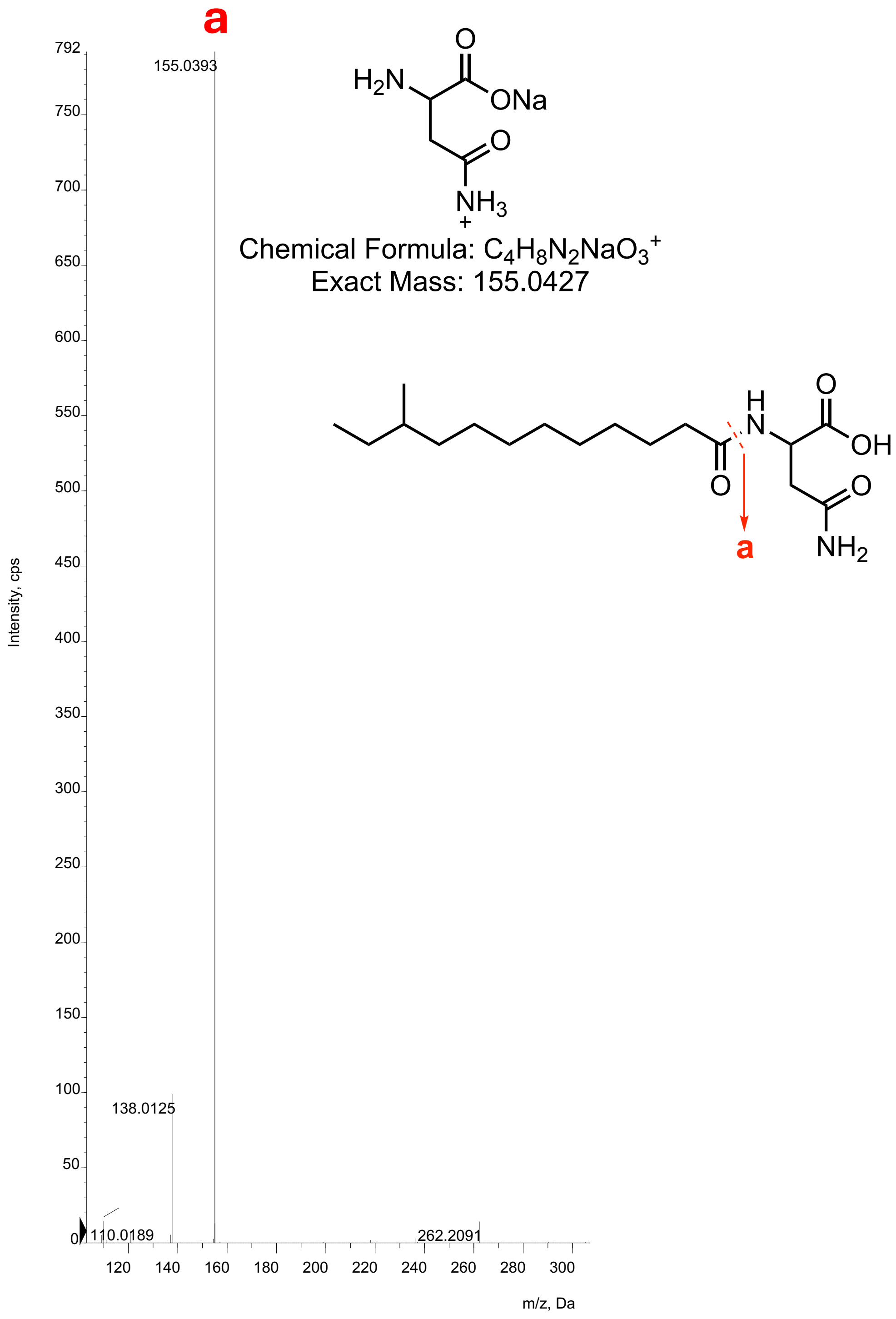

Figure S20. ^{1}H NMR spectrum of compound **4** in DMSO-d_6.

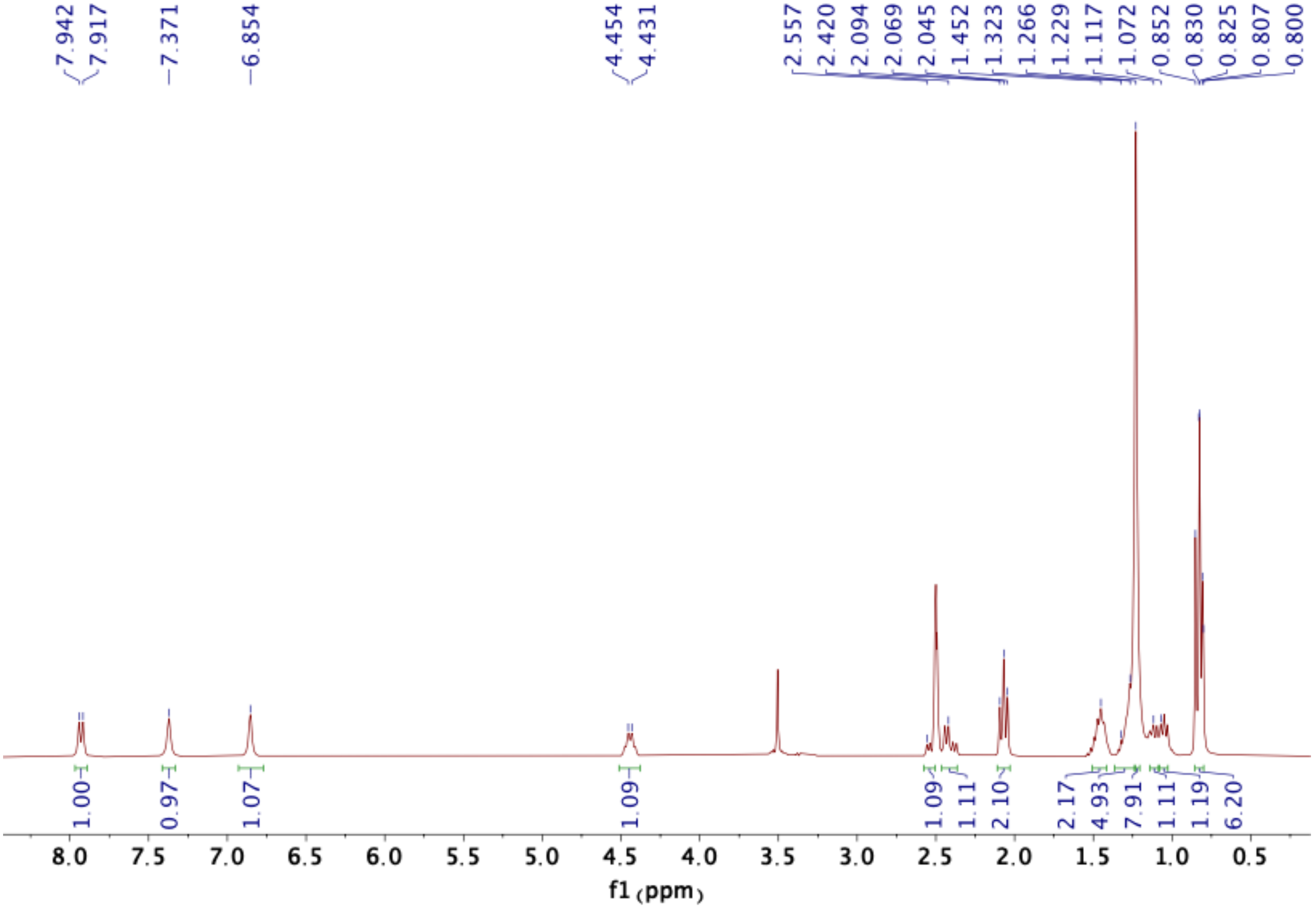

Figure S21. ^{13}C NMR spectrum of compound **4** in DMSO-d_6.

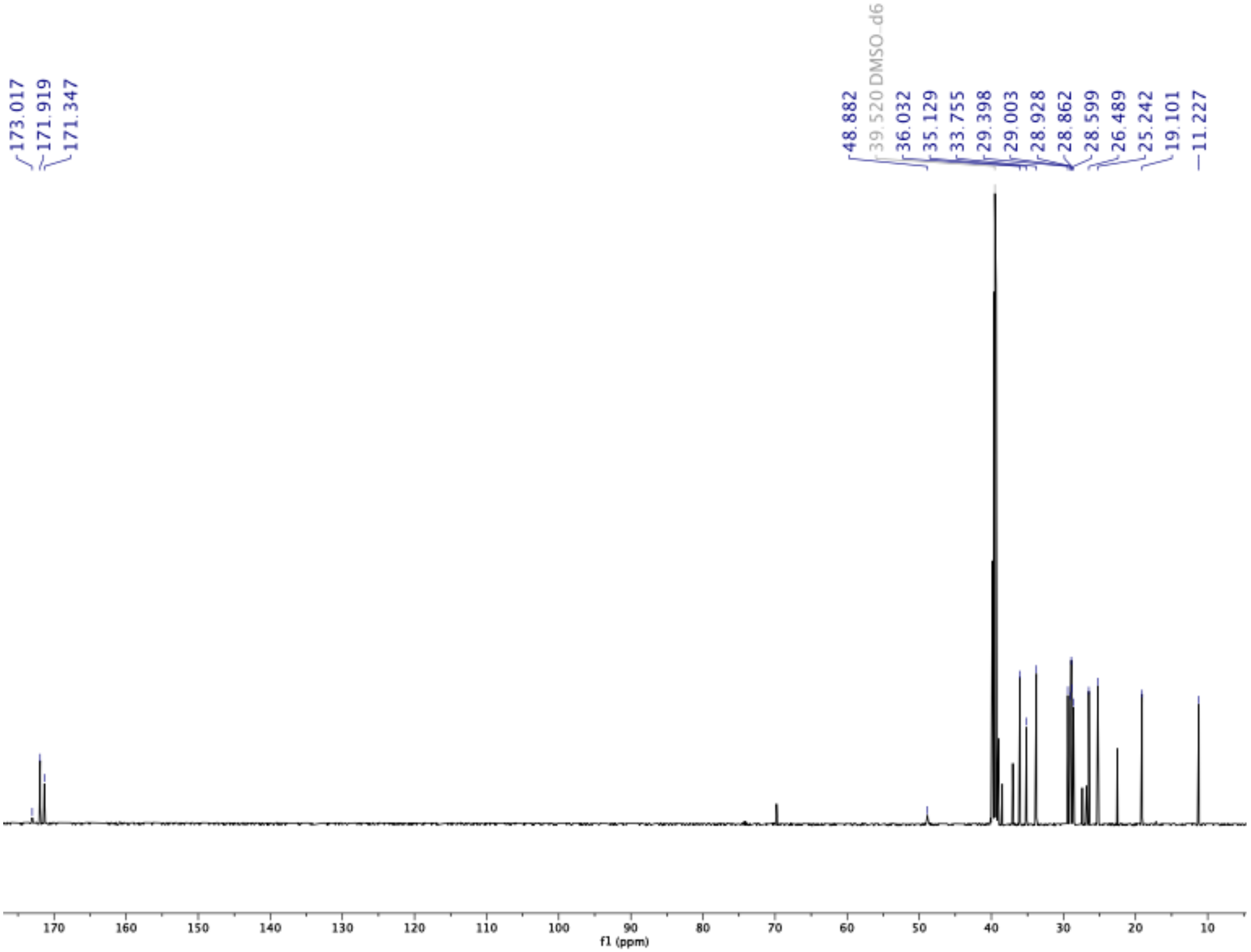

Figure S22. ^1H–^1H COSY spectrum of compound **4** in DMSO-d_6.

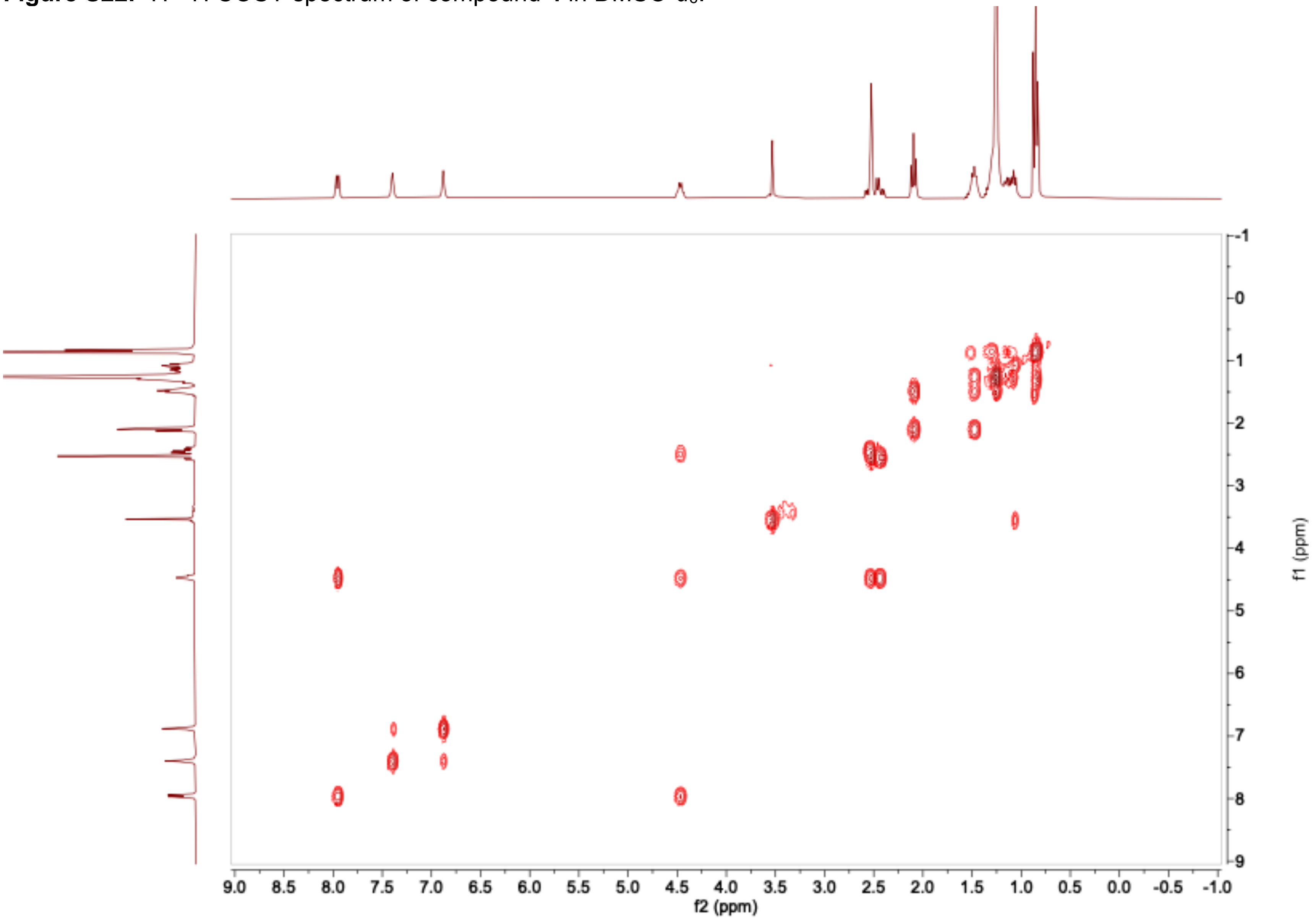

Figure S23. HSQC spectrum of compound **4** in DMSO-d_6.

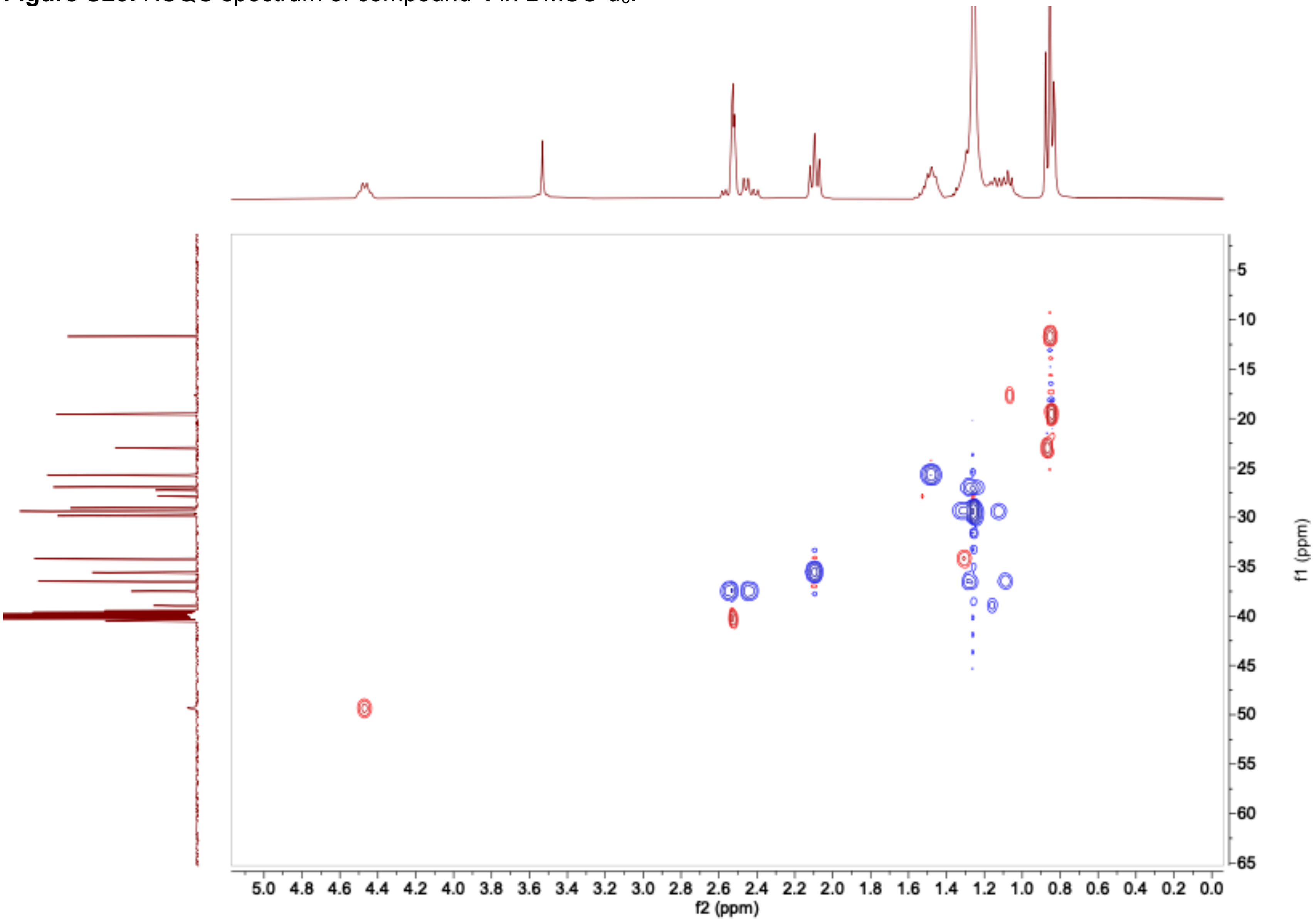

Figure S24. HMBC spectrum of compound **4** in DMSO-d_6.

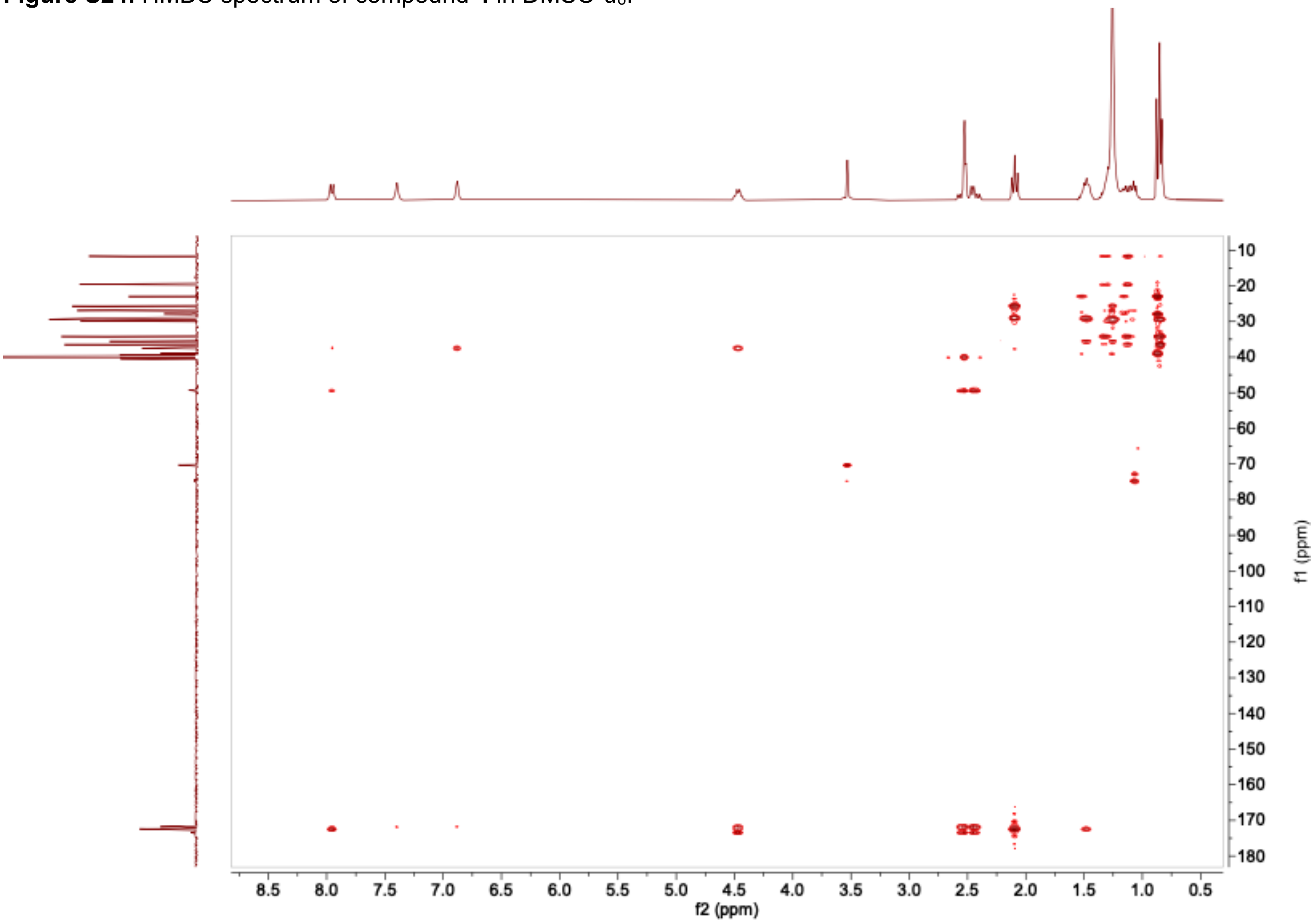

Figure S25. IR spectrum of compound **4**.

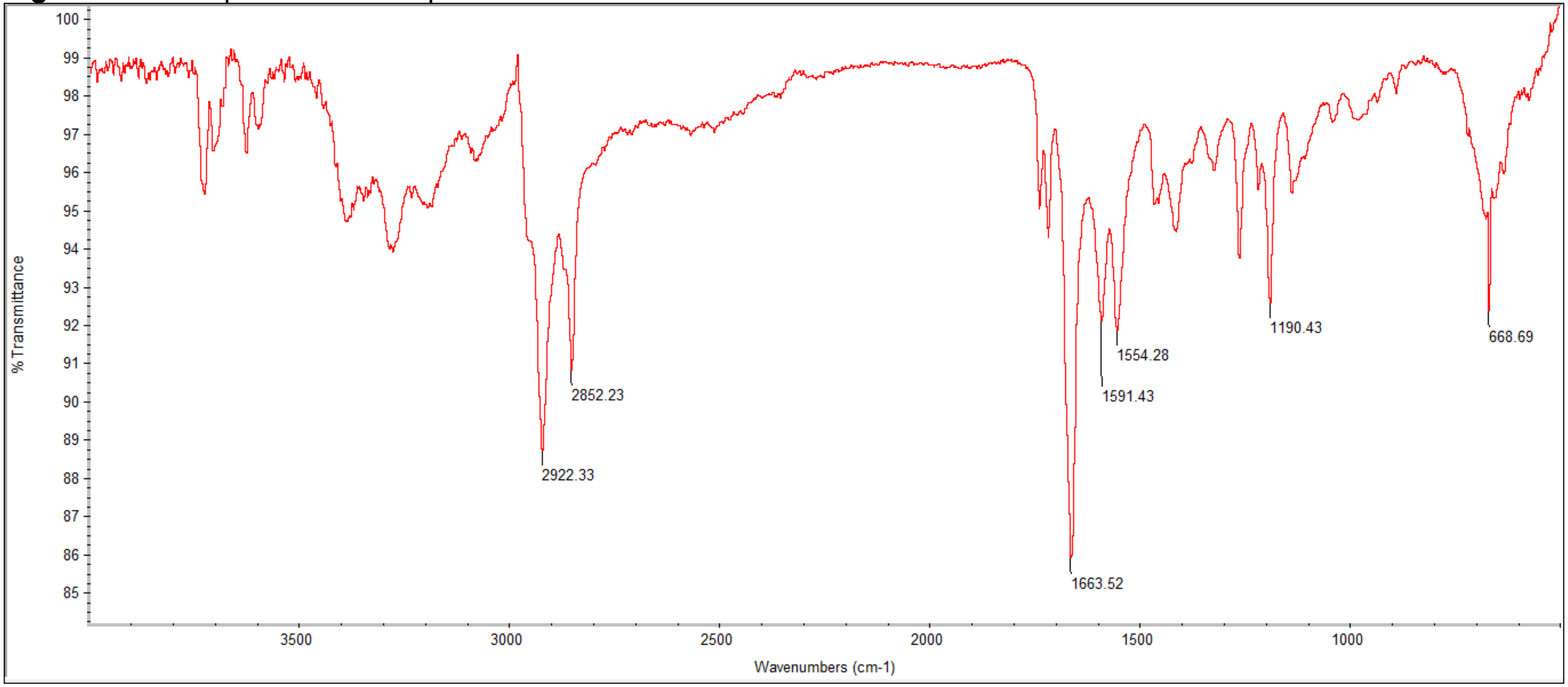

Figure S26. ^{1}H NMR spectrum of compound **5** in DMSO-d_6.

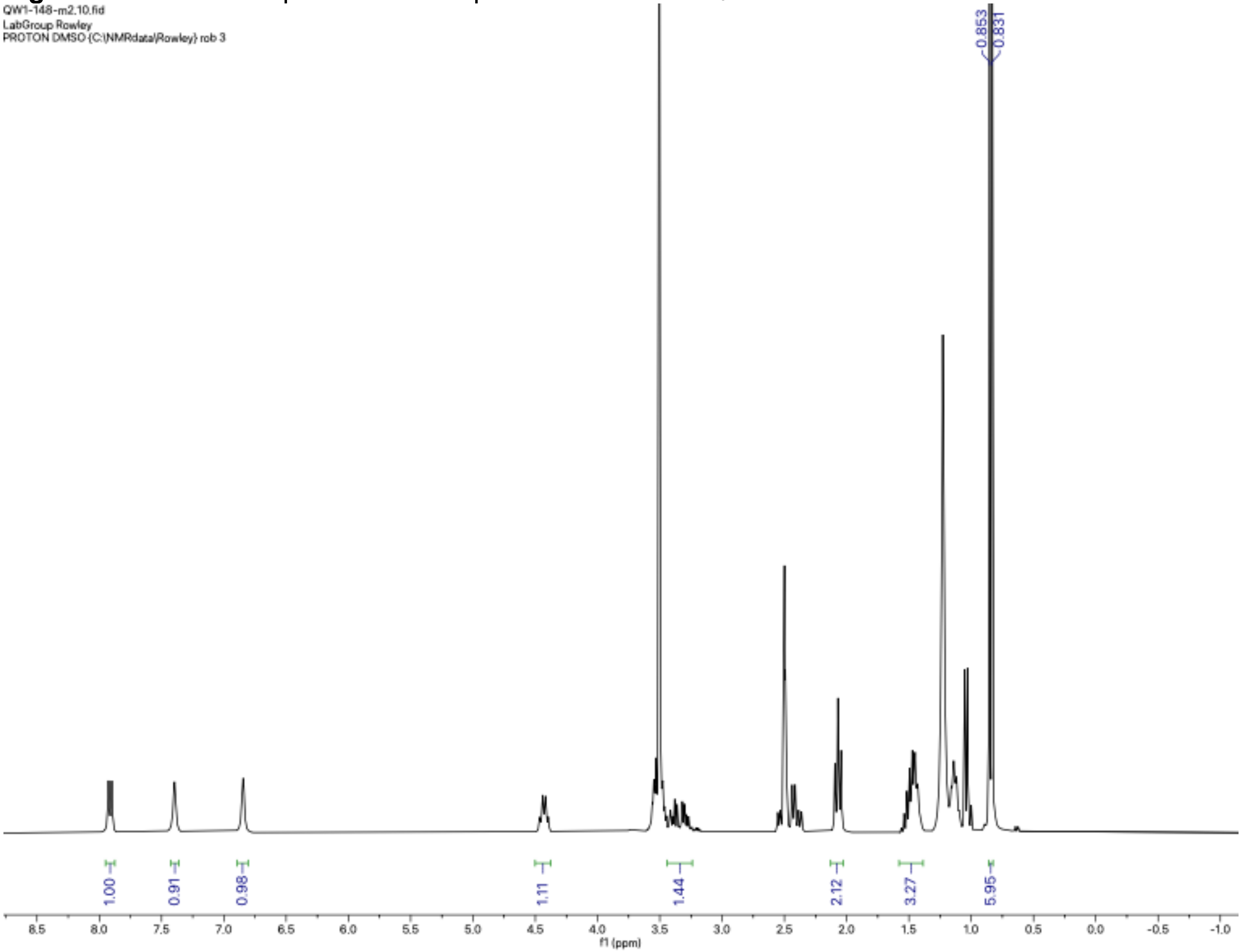

Figure S27. ^{13}C NMR spectrum of compound **5** in DMSO-d_6.

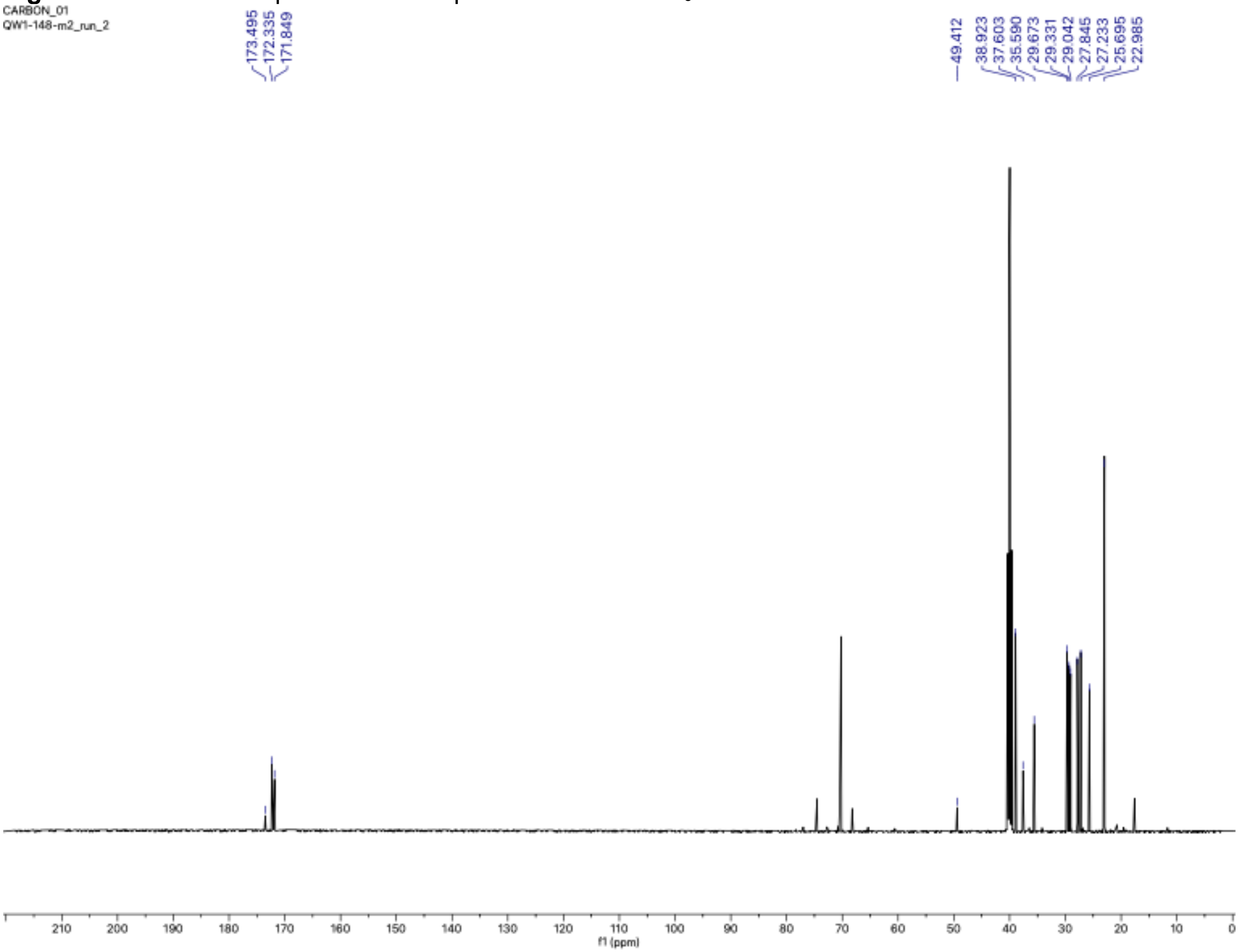

Figure S28. ^1H-^1H COSY spectrum of compound **5** in DMSO-d_6.

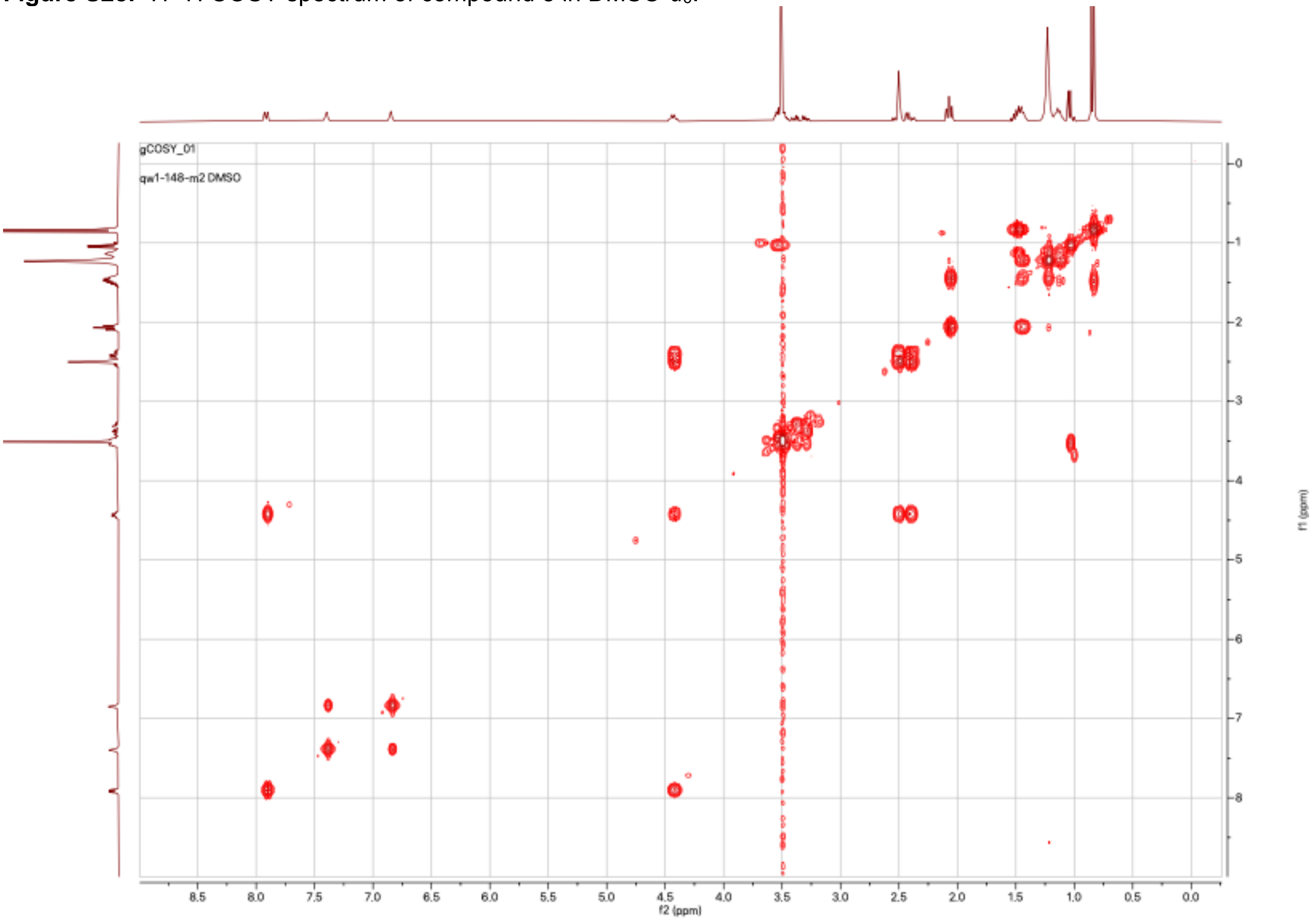

Figure S29. HSQC spectrum of compound **5** in DMSO-d_6.

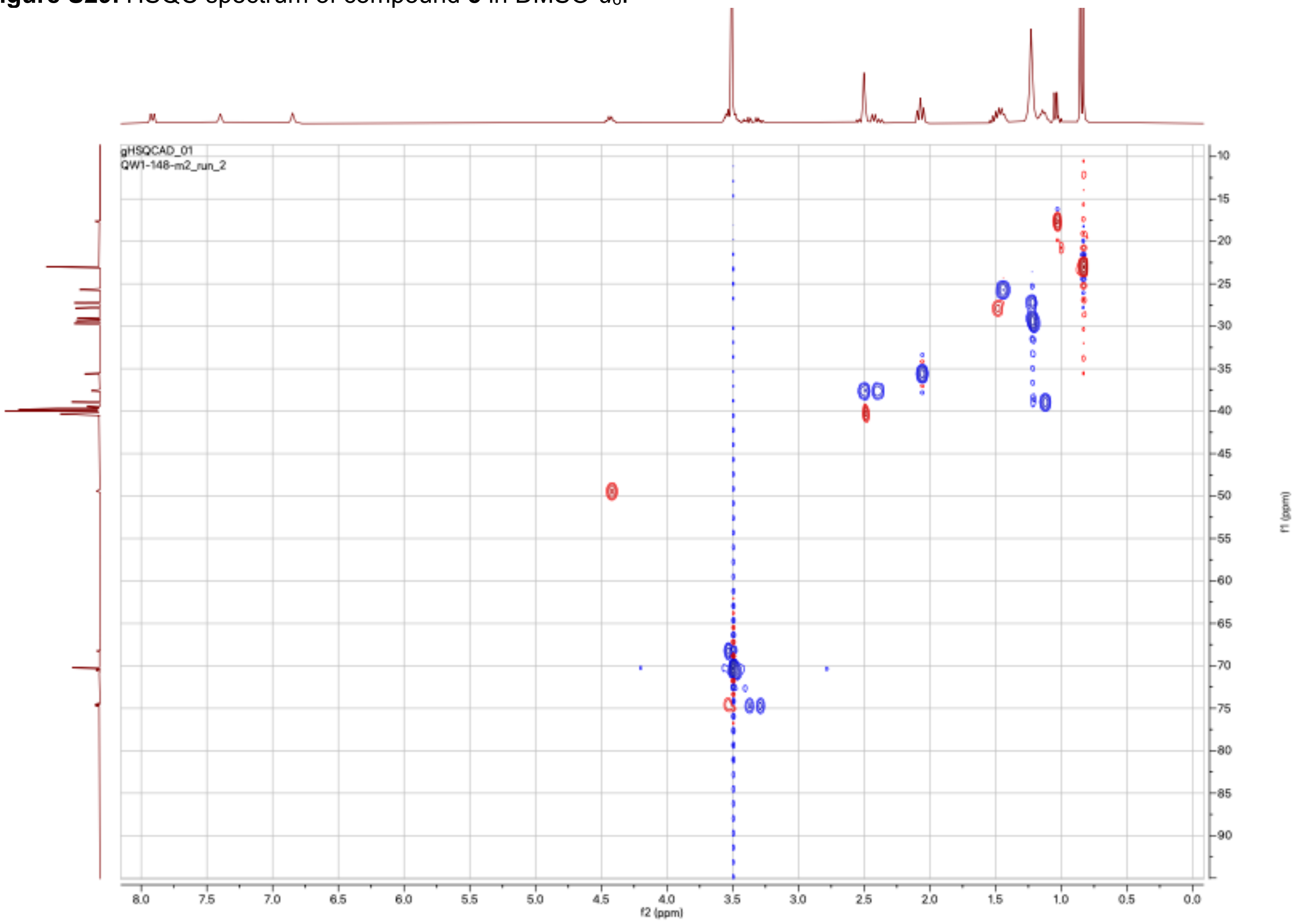

Figure S30. HMBC spectrum of compound **5** in DMSO-d_6.

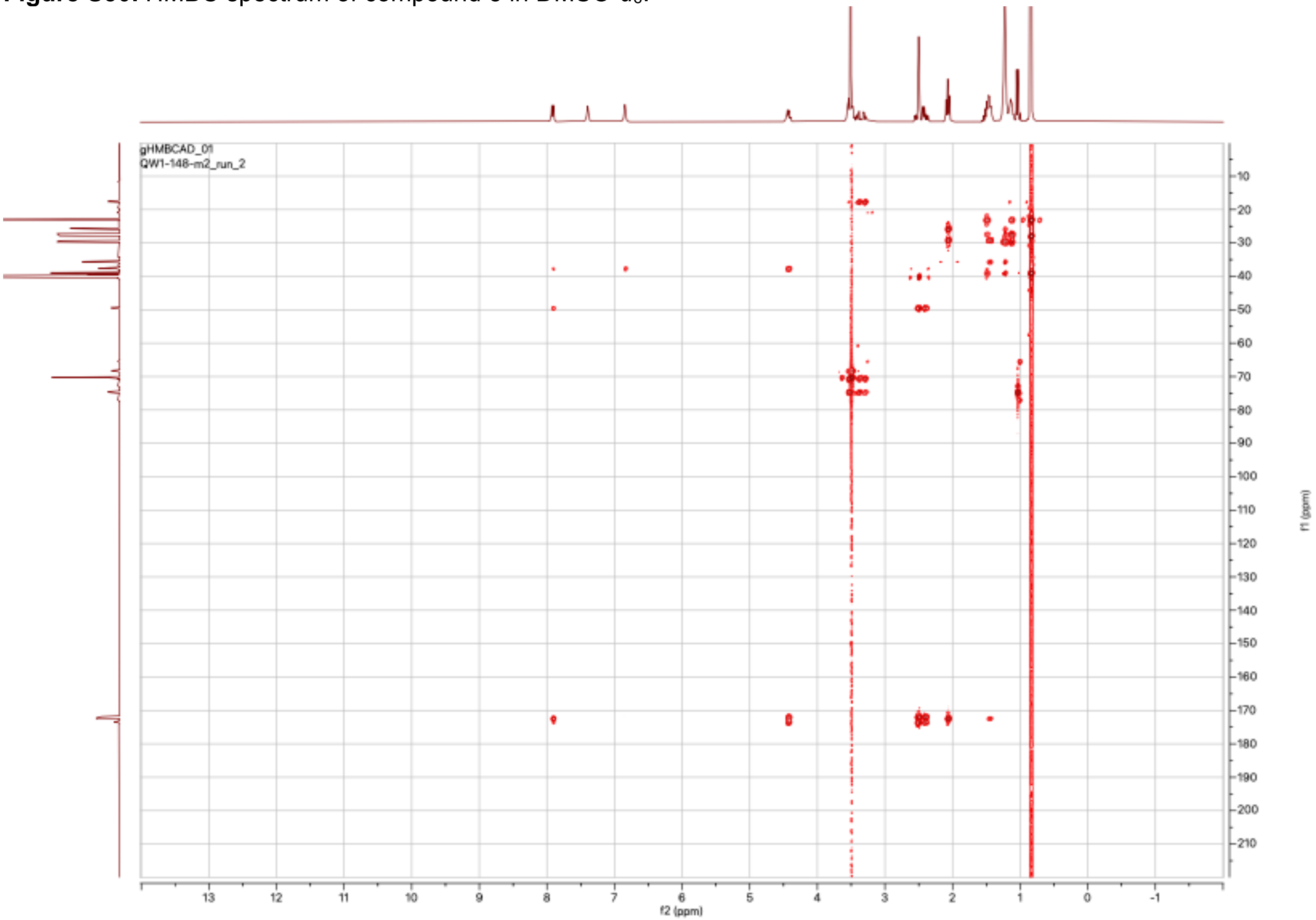

Supplement: Supplemental File — Table S1; Figures S1-S30. [file msystems.01440-25-s0001.pdf]
